# Targeting Aurora B to the Equatorial Cortex by MKlp2 Is Required for Cytokinesis

**DOI:** 10.1371/journal.pone.0064826

**Published:** 2013-06-04

**Authors:** Mayumi Kitagawa, Suet Yin Sarah Fung, Nobuyuki Onishi, Hideyuki Saya, Sang Hyun Lee

**Affiliations:** 1 Program in Cancer and Stem Cell Biology, Duke-NUS Graduate Medical School, Singapore; 2 Division of Gene Regulation, Institute for Advanced Medical Research, Keio University School of Medicine, Tokyo, Japan; Mayo Clinic, United States of America

## Abstract

Although Aurora B is important in cleavage furrow ingression and completion during cytokinesis, the mechanism by which kinase activity is targeted to the cleavage furrow and the molecule(s) responsible for this process have remained elusive. Here, we demonstrate that an essential mitotic kinesin MKlp2 requires myosin-II for its localization to the equatorial cortex, and this event is required to recruit Aurora B to the equatorial cortex in mammalian cells. This recruitment event is also required to promote the highly focused accumulation of active RhoA at the equatorial cortex and stable ingression of the cleavage furrow in bipolar cytokinesis. Specifically, in drug-induced monopolar cytokinesis, targeting Aurora B to the cell cortex by MKlp2 is essential for cell polarization and furrow formation. Once the furrow has formed, MKlp2 further recruits Aurora B to the growing furrow. This process together with continuous Aurora B kinase activity at the growing furrow is essential for stable furrow propagation and completion. In contrast, a MKlp2 mutant defective in binding myosin-II does not recruit Aurora B to the cell cortex and does not promote furrow formation during monopolar cytokinesis. This mutant is also defective in maintaining the ingressing furrow during bipolar cytokinesis. Together, these findings reveal that targeting Aurora B to the cell cortex (or the equatorial cortex) by MKlp2 is essential for the maintenance of the ingressing furrow for successful cytokinesis.

## Introduction

Cytokinesis is the final event of cell division that results in the irreversible partitioning of a mother cell into two daughter cells. It requires the localized activities of mitotic spindles and the actin cytoskeleton to activate the small GTPase RhoA at the equatorial cortex to promote the formation and ingression of the cleavage furrow [1]. This local activation of RhoA is thought to be controlled by centralspindlin, which is composed of the kinesin-6 family member MKlp1 and the Rho family GTPase RacGAP1/MgcRacGAP [2].

Another key player in cleavage furrow ingression and completion is Aurora B, the kinase component of the chromosome passenger complex (CPC) [3]. Aurora B is found at the spindle midzone and at the equatorial cortex during the meta-to-anaphase transition [4]. At the spindle midzone, Aurora B is thought to generate an anaphase phosphorylation gradient toward the cell cortex, which provides spatial information to position the cleavage furrow [5]. In contrast, the importance of cortically localized Aurora B for cytokinesis has remained elusive. Interestingly, in HeLa cells undergoing drug-synchronized monopolar cytokinesis that lack the spindle midzone [6], Aurora B but not centralspindlin localizes to the actomyosin filaments in a gap region between the end of polarized monopolar spindles and the furrowing cortical cap [7]. However, the mechanism(s) responsible for Aurora B targeting to the actomyosin filaments of the gap region as well as to the cell cortex (or the equatorial cortex in bipolar cytokinesis) is unknown. Moreover, whether this cortically targeted Aurora B is required for successful cytokinesis in mammalian cells has not been directly tested.

We show here that MKlp2, an essential mitotic kinesin for cytokinesis [8], [9], targets Aurora B to the equatorial cortex (or the cell cortex and the growing furrow in monopolar cytokinesis). Mechanistically, the cortical accumulation of MKlp2-Aurora B is accomplished by the ability of MKlp2 to bind myosin-II and actomyosin filaments. This event is required for the highly focused accumulation of active RhoA at the equatorial cortex and for efficient maintenance of the ingressing furrow in bipolar cytokinesis. Specifically, in drug-induced monopolar cytokinesis, targeting Aurora B to the cell cortex by MKlp2 is essential for cell polarization and furrow formation. Supporting this hypothesis, a MKlp2 mutant that is selectively defective in binding myosin-II does not recruit Aurora B to the cell cortex (or the equatorial cortex in bipolar cytokinesis) and does not promote cortical polarization and furrow formation during monopolar cytokinesis. Stable ingression of the cleavage furrow in bipolar cytokinesis also fails in this mutant, although the ability of MKlp2 to target Aurora B to the spindle midzone remains intact. We further demonstrate that continuous Aurora B kinase activity at the growing furrow is required for furrow propagation and completion during monopolar cytokinesis. Together, we propose that MKlp2 is an essential factor for cytokinesis that links Aurora B to the equatorial cortex (or the cell cortex and the growing furrow in monopolar cytokinesis) in mammalian cells.

## Results

### MKlp2 is Essential for the Maintenance of the Ingressing Furrow in a Partially Redundant Manner with MKlp1

**Figure 1:**
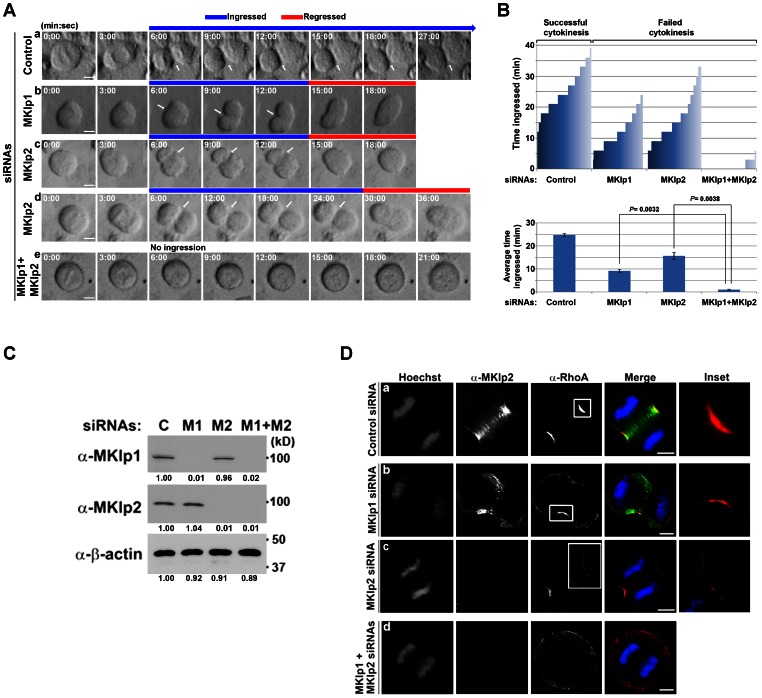
MKlp2 promotes and maintains efficient ingression of the cleavage furrow. (**A**) Synchronized HeLa cells transfected with the indicated siRNAs were subjected to time-lapse live-cell imaging. Arrows indicate the ingressed cleavage furrow. (**B**) The cytokinesis progression of cells (*n*>40) that were selected at random and the time they spent in ingression before regression is plotted (top graph). All HeLa cells depleted of MKlp1 or MKlp2 formed a cleavage furrow and ingressed followed by furrow regression and cytokinesis failure, while none of the control cells showed furrow regression after ingression. The average duration of the furrow in ingression is based on three independent experiments (total *n*>100 per condition, +/− standard deviation) (bottom graph). To determine the statistical significance of the duration of the furrow in ingression Student’s *t*-test was performed. *P* values are indicated. (**C**) Immunoblot analysis of total cell lysates from panel **A**. C: control, M1: MKlp1, M2: MKlp2. Relative band intensities to control siRNA are shown in the bottom of each panel. (**D**) Immunofluorescence analysis using asynchronously grown HeLa cells was performed at 30 h after transfection with the indicated siRNAs. Cells in anaphase are shown. Images were acquired using 3D-SIM. Insets represent the boxed areas. White bars represent 5 µm.

Although MKlp2 is essential for cytokinesis, it is still unclear how MKlp2 contributes to cytokinesis in mammalian cells. To determine the phase(s) of cytokinesis in which MKlp2 is essential, HeLa cells transfected with either control or MKlp2 siRNAs were subjected to time-lapse live-cell imaging analysis in comparison with MKlp1-depleted cells. In control cells transfected with non-silencing siRNA, the ingressed furrow was maintained until the completion of cytokinesis (Figure 1A, panel a; Figure 1B). In contrast to control cells, the furrow formed and ingressed but subsequently regressed in MKlp1-depleted cells (Figure 1A, panel b; Figure 1B, top graph). Although the duration of ingression in MKlp2-depleted cells was longer than in MKlp1-depleted cells (Figure 1B, bottom graph), the furrow formed and ingressed but was followed by furrow regression in MKlp2-depleted cells (Figure 1A, panels c, d; Figure 1B, top graph). This finding suggests that MKlp2 is required for the maintenance of the ingressing furrow at a later stage of cytokinesis compared with MKlp1 in mammalian cells. Remarkably, however, co-depletion of MKlp1 and MKlp2 largely inhibited furrow ingression (Figure 1A, panel e; Figure 1B), suggesting that MKlp2 acts during an early stage of furrow ingression in the absence of MKlp1 in mammalian cells. This result was not due to incomplete depletion of MKlp1 or MKlp2 proteins as determined by immunoblot analysis (Figure 1C), indicating that MKlp1 and MKlp2 function in partially redundant pathways for furrow ingression.

RhoA is required for furrow formation and stable ingression. Notably, the RhoA zone was tightly focused at the equatorial cortex in control cells, whereas the zone was more diffuse in MKlp2-depleted cells (Figure 1D). Moreover, the maximum intensity projection of serial optical sections through the equatorial cortex revealed that the RhoA zone became diffuse and more unevenly distributed at the equatorial cortex in MKlp2-depleted cells compared with control cells (Figure S1A). As co-depletion of MKlp1 and MKlp2 largely inhibited furrow ingression (Figure 1A, panel e), it also eliminated the RhoA zone from the equatorial cortex (Figure 1D). This result indicates that MKlp2 is responsible for focusing active RhoA at the equatorial cortex. Specifically, the depletion of either MKlp alone did not significantly affect other MKlp levels (Figure 1C), and the depletion of MKlp2 using different siRNAs did not significantly affect the levels of centralspindlin or CPC components (Figure S1B; siRNA #3 was used in rescue experiments). Moreover, in HeLa cell lines engineered to express Flag-tagged siRNA-resistant MKlp2 at endogenous levels upon doxycycline (Dox)-treatment, the RhoA zone was focused more tightly at the equatorial cortex compared with non-induced cells (Figure S1C, S1D). Notably, the total levels of RhoA within the equatorial cortex were similar between control and MKlp2-depleted cells (data not shown), although the RhoA zone was less focused, indicating the unlikelihood that MKlp2 is involved in RhoA activation. Together, our data suggest that MKlp2 promotes the polarized high accumulation of RhoA at the equatorial cortex, which is required for maintaining stable furrow ingression.

### MKlp2 Localizes to the Equatorial Cortex via its Ability to Bind Myosin-II and Actomyosin Filaments and is Required for Maintaining the Ingressing Furrow

**Figure 2:**
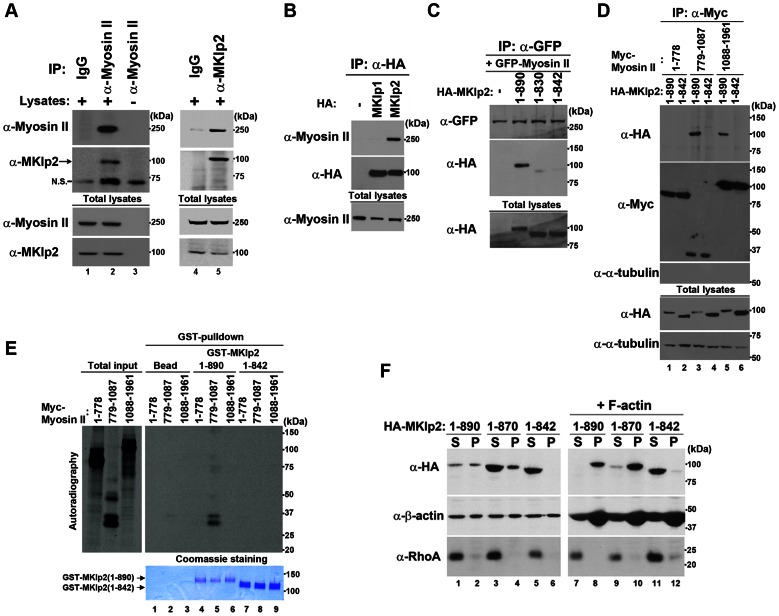
MKlp2 is a novel binding partner of myosin-II *in vivo* and *in vitro*. (**A**) Asynchronously growing HeLa cells were harvested and subjected to immunoprecipitation analysis using antibodies for pre-immune control IgG (lanes 1, 4), myosin-II (lanes 2, 3), and MKlp2 with (lane 5) or without (lane 3) HeLa cell lysates. Note that the 100 kDa band is specific for MKlp2 (lane 2) and not caused by α-Myosin-II antibodies used for immunoprecipitation as it is not detected in lane 3. N.S. indicates non-specific. (**B**–**D**) Asynchronously growing HeLa cells were transfected with the indicated expression plasmids, and 24 h after transfection, HeLa cell lysates expressing the indicated MKlp2 or myosin-II proteins were subjected to immunoprecipitation with the indicated antibodies. (**E**) Autoradiography of *in vitro*-translated Myc-Myosin-II precipitates with the indicated GST-MKlp2 proteins using GST-pulldown analysis (bottom, visualized with Coomassie Blue staining). Overall, 10% of the input for total *in vitro*-translated product is shown. (**F**) F-actin binding assay. Asynchronously growing HeLa cells were transfected with the indicated expression plasmids, and 24 h after transfection, HeLa cell lysates expressing the indicated HA-MKlp2 proteins were supplemented without (lanes 1-6) or with *in vitro*-polymerized recombinant F-actin (lanes 7-12) and subjected to ultracentrifugation. Supernatant (S) and pellet (P) fractions were subjected to immunoblot analysis.

Endogenous (Figure 1D, panel a) and Dox-induced Flag-MKlp2 (Figure S1D) accumulated at the equatorial cortex in addition to the spindle midzone, suggesting that MKlp2 may function in furrow ingression at the equatorial cortex. To determine the potential MKlp2-mediated mechanisms(s) involved in furrow ingression at the equatorial cortex, we searched for binding partner(s) of MKlp2 by performing affinity purification of stably expressed Flag-MKlp2 using the HEK293 cell line. Using mass spectrometry analysis, non-muscle myosin-II-A (24 unique peptides) and myosin-II-B (30 unique peptides), herein referred to as myosin-II, were identified in immunoprecipitates from Flag-MKlp2 but not in control cells (data not shown). Indeed, using immunoprecipitation analysis, endogenous MKlp2 and myosin-II were precipitated together in a reciprocal manner (Figure 2A). Notably, endogenous myosin-II was co-precipitated with HA-tagged MKlp2 but not MKlp1 (Figure 2B). Moreover, compared with full-length HA-MKlp2(1-890), HA-MKlp2(1-842) failed to bind GFP-tagged myosin-II (Figure 2C). Conversely, HA-MKlp2(1-890) bound strongly to the neck domain (a.a. 779-1087) and weakly to the tail domain (a.a. 1088-1961) of myosin-II (Figure 2D). Notably, the head domain (a.a. 1-778) of myosin-I, which is responsible for binding filamentous actin, was not found to interact with MKlp2, suggesting that the interaction between MKlp2 and myosin-II was not due to the ability of myosin-II to bind filamentous actin. Specifically, HA-MKlp2(1-842) did not bind myosin-II (Figure 2D); however, the ability of HA-MKlp2(1-842) to bind microtubules, Aurora B and Plk1 was intact and comparable to HA-MKlp2(1-890) (Figure S2). Furthermore, the *in vitro*-translated neck domain of Myc-tagged myosin-II bound recombinant GST-MKlp2(1-890) but not GST-MKlp2(1-842) (Figure 2E), suggesting that MKlp2 is a binding partner of myosin-II *in vivo* and *in vitro*. Consistent with its ability to bind myosin-II, the majority of HA-MKlp2(1-890) and HA-MKlp2 (1-870) co-sedimented with *in vitro* polymerized F-actin but not MKlp2(1-842) (Figure 2F), suggesting that MKlp2 forms a complex with actomyosin filaments.

**Figure 3:**
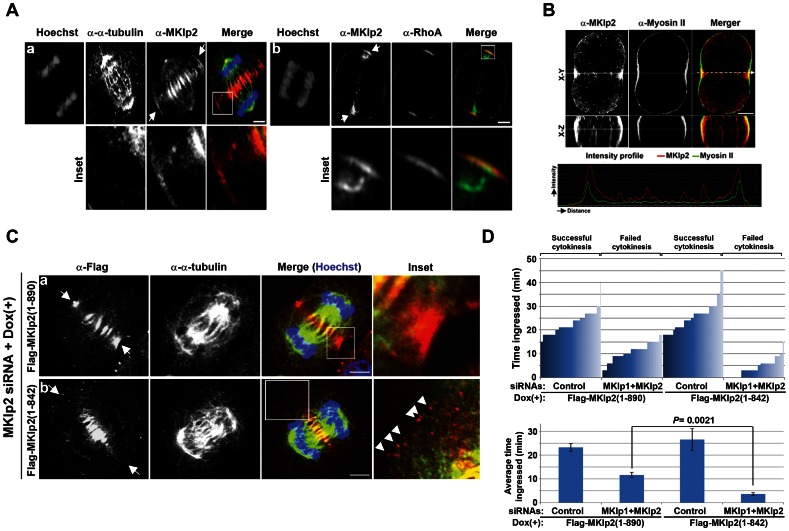
MKlp2 localizes to the equatorial cortex prior to furrow ingression and promotes furrow ingression. (**A**, **B**) Immunofluorescence analysis. Asynchronously growing HeLa cells were fixed with ice-cold methanol (**A**, panel a; **B**) or 10% TCA (**A**, panel b) and stained with the indicated antibodies. Arrows indicate the equatorial cortex. (**B**) X-Z shows a cross-section at the equator (white arrow) with an intensity profile of the corresponding section (bottom). (**C**) Images of Dox-induced Flag-MKlp2 in MKlp2-depleted HeLa cells using siRNA transfection. Dox-inducible HeLa cells were transfected for 4 h with siRNA to depleted endogenous MKlp2. Subsequently, cells were treated with Dox for 20 h before fixing with ice-cold methanol. Flag-MKlp2(1-890) accumulated at the equatorial cortex as indicated with arrows (panel a), whereas Flag-MKlp2(1-842) only showed punctate staining patterns close to the equatorial cortex (panel b, arrow heads in inset). However, both proteins were comparably localized to the spindle midzone. For panels **A** and **C**, images were acquired using confocal microscopy. For panel **B**, images were acquired using 3D-SIM. Insets represent the boxed areas. White bars represent 5 µm. (**D**) HeLa cells were transfected with non-silencing control siRNA or co-transfected with MKlp1 and MKlp2 siRNAs after the first thymidine block and were treated with Dox during the second thymidine block (see Materials and Methods). The cells were synchronously released from the G_1_/S boundary and subjected to time-lapse live-cell imaging. The cytokinesis progression of cells that were selected at random, and the time they spent in ingression before regression is plotted (top graph). The average duration of furrow in ingression is based on three independent experiments (total *n*>100 per condition, +/− standard deviation) (bottom graph). To determine the statistical significance of the duration of furrow in ingression, Student’s *t*-test was performed. *P* values are indicated.

Next, we determined whether these results obtained by the biochemical analysis explained MKlp2 localization at the equatorial cortex. Endogenous MKlp2 localized to the equatorial cortex (Figure 3A) together with RhoA (panel b) and myosin-II (Figure 3B) prior to cleavage furrow ingression as shown by immunofluorescence analysis using HeLa cells. Dox-induced Flag-MKlp2(1-890) completely co-localized with Aurora B at the cell equator (Figure S3A, S3B), supporting the hypothesis that Aurora B is the mitotic cargo of MKlp2 [9]. Moreover, similar to endogenous MKlp2, Flag-MKlp2(1-890) accumulated at the equatorial cortex and the spindle midzone (Figure 3C, panel a). In contrast, Flag-MKlp2(1-842) selectively failed to accumulate at the equatorial cortex, while it efficiently localized to the spindle midzone (Figure 3C, panel b). This result is consistent with the finding that Flag-MKlp2(1-842) was selectively defective in binding myosin-II but not other known interacting partners of MKlp2 (Figure S2). Interestingly, the RacGAP1 centralspindlin component comparably localized to the spindle midzone in cells expressing Flag-MKlp2(1-890) and Flag-MKlp2(1-842) (Figure S3C, S3D). Together, these results suggest that the MKlp2:myosin-II interaction is likely required for the accumulation of MKlp2 at the equatorial cortex.

Flag-MKlp2(1-842) was selectively defective in accumulating at the equatorial cortex; therefore, we tested the importance of MKlp2 at the equatorial cortex to maintain furrow ingression. To address this idea, endogenous MKlp1 and MKlp2 were co-depleted to inhibit furrow ingression as shown in Figure 1A (panel e). Subsequently, these cells were treated with Dox to induce Flag-MKlp2 and were subjected to time-lapse live-cell imaging. In either Flag-MKlp2(1-890)- or Flag-MKlp2(1-842)-induced HeLa cells transfected with non-silencing control siRNA, the furrow was efficiently ingressed and maintained until the completion of cytokinesis (Figure 3D, top graph). Interestingly, a majority of Flag-MKlp2(1-890)-induced HeLa cells co-depleted of MKlp1 and MKlp2 began the process of furrow ingression but subsequently regressed (Figure 3D, top graph) as observed in MKlp1-depleted cells (Figure 1B), suggesting that Dox-induced Flag-MKlp2(1-890) was functional for maintaining furrow ingression. Under this condition, however, Flag-MKlp2(1-842) largely failed to initiate and maintain the ingressing furrow (Figure 3D, top graph) with the average duration of furrow ingression shorter compared with cells expressing Flag-MKlp2(1-890) (Figure 3D, bottom graph). Interestingly, Dox-induced Flag-MKlp2(1-842) in co-depleted MKlp1 and MKlp2 cells largely failed to initiate and maintain the ingressing furrow; however, Flag-MKlp2(1-842) partially rescued the mutant phenotype compared with co-depleted cells without Flag-MKlp2(1-842). This finding suggests that MKlp2 may have functions independent of the Myosin II interaction, which may possibly occur at the central spindles. Nevertheless, these results reveal that MKlp2 contributes to maintaining the ingressing furrow at the equatorial cortex during bipolar cytokinesis.

### MKlp2 is Essential for Aurora B Localization at the Cell Cortex and for Cell Polarization during Monopolar Cytokinesis

**Figure 4:**
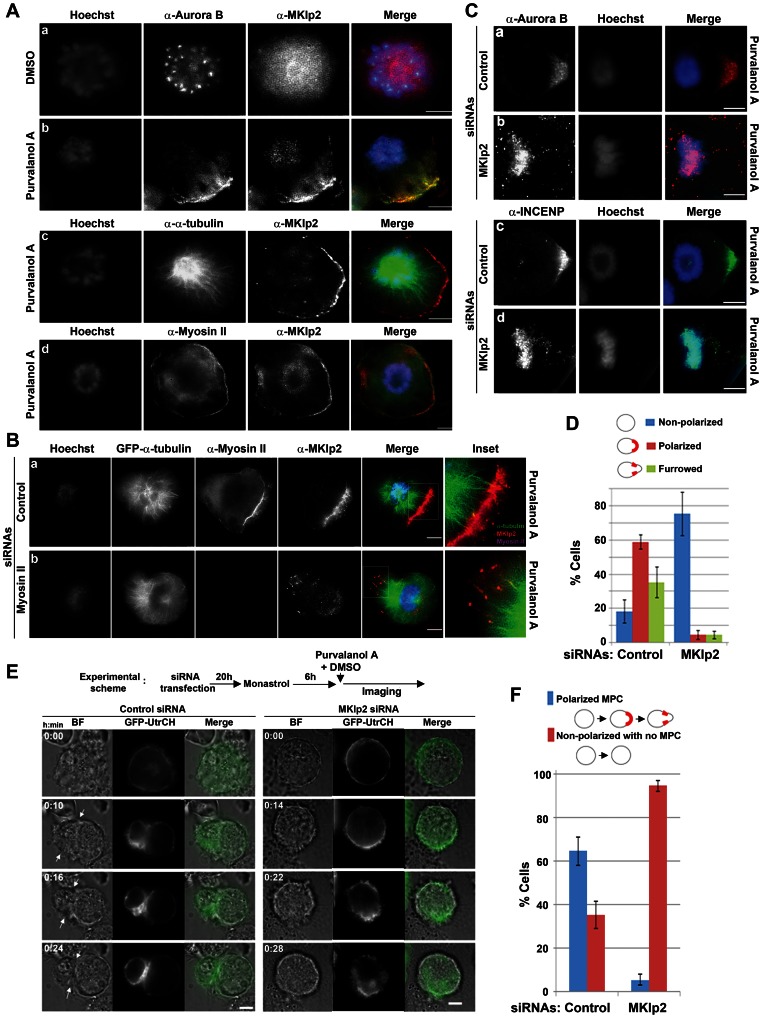
MKlp2 localizes to the growing furrow in a myosin-II-dependent manner, and it is required for cell polarization during monopolar cytokinesis. (**A**) Immunofluorescence analysis of HeLa cells before (panel a) or during monopolar cytokinesis (panels b-d). Asynchronously growing HeLa cells were treated with monastrol for 6 h and then treated with Purv A or DMSO for 15 min before fixation in ice-cold methanol. (**B, C**) HeLa cells were transfected with the indicated siRNAs for 20 h before the addition of monastrol, and monopolar cytokinesis was induced as described in panel **A**. For panel **B**, HeLa cells stably expressing GFP-α-tubulin were used. For panels **A–C**, images were acquired using 3D-SIM. (**D**) The average percentages calculated based on three independent experiments using non-polarized, polarized (determined by cortically localized Aurora B from panel **C**) or furrowed cells (determined by myosin-II) are shown with error bars (*n*>100 cells per condition). (**E**) Time-lapse live-cell imaging. HeLa cells were transfected with the indicated siRNAs together with the vectors encoding GFP-UtrCH to monitor cortical changes and were subjected to monopolar cytokinesis. Arrows indicate the site where a typical polarized furrow was formed and progressed. (**F**) Monopolar cells (from panel **E**) were scored as cells that completed monopolar cytokinesis (polarized MPC) or failed without polarizing the cortex (non-polarized with no MPC). The average percentages based on three independent experiments (total *n*>100 per condition, +/− standard deviation) are shown. Images were acquired using 2D-SIM. White bars represent 5 µm.

The close proximity of the spindle midzone and the equatorial cortex in the ingressing furrow during bipolar cytokinesis creates difficultly in precisely determining the role of MKlp2 within this interface. To better address the issue, we induced drug-synchronized monopolar cytokinesis [6] where Aurora B but not centralspindlin localizes to the actomyosin filaments in a gap region between the end of polarized monopolar spindles and the furrowing cortical cap [7]. In monastrol-arrested monopolar HeLa cells, the majority of MKlp2 and Aurora B was present in the cytoplasm and at the centromeres, respectively (Figure 4A, panel a). However, when these cells were forced to undergo monopolar cytokinesis caused by treatment with the potent Cdk1 inhibitor purvalanol A (Purv A), endogenous MKlp2 efficiently co-localized with Aurora B at the polarized furrowing cortex (Figure 4A, panel b). Moreover, MKlp2 co-localized with myosin-II adjacent to the polarized cortex (Figure 4A, panel d), but it was clearly distinctive from the polarized monopolar spindles (panel c). Using HeLa cells stably expressing GFP-α-tubulin and super-resolution fluorescence microscopy, MKlp2 was shown to be localized to the ends of polarized monopolar spindles and extended to the polarized actomyosin filaments at the growing furrow (Figure 4B, panel a), indicating that MKlp2 may be involved in linking actomyosin filaments at the growing furrow with polarized monopolar spindles. In contrast, siRNA-mediated depletion of myosin-II (Figure S4A) markedly suppressed the localization of MKlp2 at the cell cortex but not at the ends of monopolar spindles (Figure 4B, panel b).Of note, it is possible that since no polarized cortical structures might form in myosin-II-depleted cells, which may prevent MKlp2 from localizing to the cell cortex. Nevertheless, our results indicate that myosin-II is required for MKlp2 to localize to the actomyosin filaments at the growing furrow.

Conversely, depletion of MKlp2 did not affect the cortical localization of myosin-II as well as its protein levels (Figure S4B, S4C). The cortical localization of Aurora B and INCENP was completely inhibited in MKlp2-depleted cells; however, they were efficiently recruited to the growing furrow in control cells (Figure 4C). Instead, Aurora B and INCENP largely remained on chromosomes in MKlp2-depleted cells, confirming that MKlp2 is required for removing the CPC from anaphase chromatin during bipolar cytokinesis [9]. Furthermore, as determined by immunofluorescence analysis, depletion of MKlp2 blocked cortical polarization and inhibited subsequent furrow formation (Figure 4D). This result was also confirmed using time-lapse live-cell imaging analysis showing that MKlp2-depleted cells neither stably polarized the cell cortex nor formed a furrow, while control cells efficiently underwent these processes (Figure 4E, 4F). Interestingly, in these MKlp2-depleted cells, the tip of the monopolar spindle, which was visualized using spindle end-tracking GFP-EB1 in MKlp2-depleted cells, was able to contact the cell cortex (Figure S4C). This result suggests that the failure in polarization was not simply due to a defect in the polymerization of the monopolar spindles that ultimately prevents contact with the cell cortex. Together, our data reveal that MKlp2 is required for cell polarization and subsequent furrow formation in monopolar cytokinesis.

### MKlp2 Recruits Aurora B to the Cell Cortex in Order to Promote Cell Polarization and Furrow Formation during Monopolar Cytokinesis

Next, we determined whether MKlp2 actually targets Aurora B to the cell cortex and subsequently to the growing furrow during monopolar cytokinesis. Furthermore, we wished to determine whether this event is important for polarization and furrow formation. However, siRNA-mediated depletion of MKlp2 alone resulted in the retention of Aurora B on chromosomes, making it impossible to directly evaluate the role of MKlp2 and Aurora B at the cell cortex. Moreover, inhibiting the kinase activity of Aurora B prior to its recruitment to the cell cortex was not an ideal method to determine this issue because such untimely inhibition also causes the mislocalization of other essential factors that are involved in furrow formation (e.g., centralspindlin) from the monopolar spindles as well as the cell cortex [7], [10], [11].

**Figure 5:**
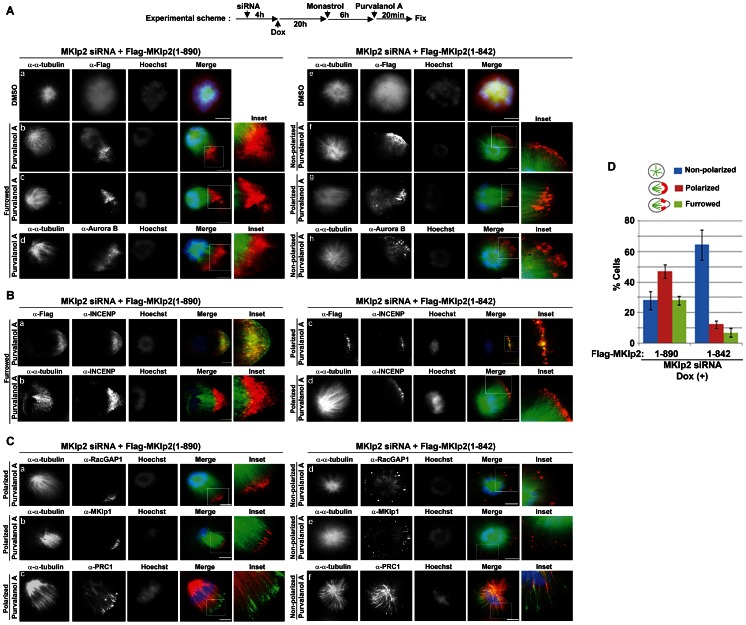
Recruiting Aurora B to the cell cortex and the growing furrow by MKlp2 is required for stable polarization and furrow formation during monopolar cytokinesis. (**A–C**) Immunofluorescence analysis of Dox-inducible HeLa cells undergoing monopolar cytokinesis. Flag-MKlp2 expression was induced with Dox for 20 h after the transfection of MKlp2 siRNA. After Dox-treatment, cells were treated with monastrol for 6 h and then treated with Purv A or DMSO for 20 min before fixation in ice-cold methanol. (**A, B**) Flag-MKlp2(1-890) localized to the cell cortex and at the growing furrow together with Aurora B (A, panels b-d) and INCENP (**B**, panels a, b), but Flag-MKlp2(1-842) only localized to the ends of monopolar spindles together with Aurora B (**A**, panels f-h) and INCENP (**B**, panels c, d). (**C**) In contrast to Aurora B and INCENP in panels **A** and **B**, centralspindlin and PRC1 were efficiently localized to the ends of monopolar spindles in cells expressing Flag-MKlp2(1-890) and MKlp2(1-842). This finding indicates that Flag-MKlp2(1-842) is selectively defective in targeting Aurora B and INCENP (thus, most likely the CPC) from the monopolar spindles to the cell cortex. (**D**) The average percentages based on three independent experiments of non-polarized, polarized (determined by the direction of Flag-MKlp2 and the monopolar spindles towards the cell cortex) or furrowed cells (determined by the presence of Flag-MKlp2 and myosin-II in the furrow) expressing Flag-MKlp2 (*n*>100 per condition, +/− standard deviation) are shown.

Therefore, to avoid these complications, we again utilized the rescue assay system consisting of Dox-inducible siRNA-resistant Flag-MKlp2 after knockdown of endogenous MKlp2 in HeLa cells. Similar to the case of endogenous MKlp2 (Figure 4A), upon induction of monopolar cytokinesis by Purv A treatment, Dox-induced Flag-MKlp2(1-890) relocated to the polarized furrow together with Aurora B (Figure 5A, panels b-d; Figure S5A, panel a). When these cells were fixed at different time points after Purv A treatment, Flag-MKlp2(1-890) gradually moved to the cell cortex (Figure S6A, panels b, c) and subsequently to the gap region at the growing furrow (panel d). In contrast, Flag-MKlp2(1-842) defective in binding myosin-II was only able to accumulate at the ends of either non-polarized or polarized monopolar spindles in MKlp2-depleted HeLa cells and failed to accumulate at the cell cortex (Figure 5A, panels f, g; Figure S6A, panels e-h). This result is consistent with the inability of Flag-MKlp2(1-842) to localize to the equatorial cortex but not the spindle midzone in bipolar cells (Figure 3C). Moreover, Aurora B was also found at the ends of monopolar spindles together with Flag-MKlp2(1-842) but not at the cell cortex (Figure 5A, panel h; Figure S5A, panel b). Similar to Aurora B, Flag-MKlp2(1-890) moved together with INCENP to the gap region at the growing furrow (Figure 5B, panels a, b). In contrast, Flag-MKlp2(1-842) was only able to localize INCENP at the ends of monopolar spindles in MKlp2-depleted HeLa cells (Figure 5B, panels c, d), indicating that MKlp2 is responsible for localizing both Aurora B and INCENP (thus, most likely the CPC) to the cell cortex. Specifically, in our rescue assay, other essential factors for furrow formation such as centralspindlin and PRC1 were correctly localized to the ends of monopolar spindles towards the cell cortex (Figure 5C). Together, these results strongly suggest that Flag-MKlp2(1-842) is selectively defective in targeting the CPC from the monopolar spindles to the cell cortex and that this deficiency is likely specific to the CPC without affecting the localization of other essential furrow-inducing factors during monopolar cytokinesis.

Therefore, using this system, we determined whether MKlp2-Aurora B at the cell cortex is important for polarization and furrow formation during monopolar cytokinesis. Quantification of the number of cells undergoing monopolar cytokinesis showed that Flag-MKlp2(1-890) efficiently rescued polarization and furrow formation in MKlp2-depleted cells (Figure 5D). In contrast, the number of polarized cells with a furrowing cortex was markedly decreased in MKlp2-depleted cells expressing Flag-MKlp2(1-842) (Figure 5D). Consistent with this result, RhoA was also inefficiently polarized in cells expressing Flag-MKlp2(1-842) compared with Flag-MKlp2(1-890) (Figure S6B). This result is similar to the diffused RhoA zone at the equatorial cortex observed in MKlp2-depleted cells (Figure 1D; Figure S1A). Moreover, time-lapse live-cell analysis using MKlp2-depleted HeLa cells expressing GFP-α-tubulin and siRNA-resistant mCherry-MKlp2 showed that mCherry-MKlp2(1-890) localized to the growing furrow leading to furrow completion (Figure S7A, S7D). In contrast, mCherry-MKlp2(1-842) only localized to the ends of monopolar spindles and failed to support monopolar cytokinesis (Figure S7B, S7D). Notably, it is unlikely that furrowing in general is required for MKlp2 and Aurora B to localize to the cell cortex and to the growing furrow because blocking furrow activity by brief treatment with the myosin-II inhibitor (-)-Blebbistatin prior to Purv A treatment did not prevent Flag-MKlp2(1-890) and Aurora B from localizing to the cell cortex (Figure S8, panels b, c). Under this condition, Flag-MKlp2(1-842) and Aurora B were also observed at the ends of monopolar spindles (Figure S8, panels d, e). Together, we conclude that a major role of MKlp2-Aurora B at the cell cortex involves promoting efficient cell polarization and subsequent furrow formation during monopolar cytokinesis.

### Continuous Aurora B kinase Activity at the Growing Furrow is Required for Furrow Propagation and Completion

**Figure 6:**
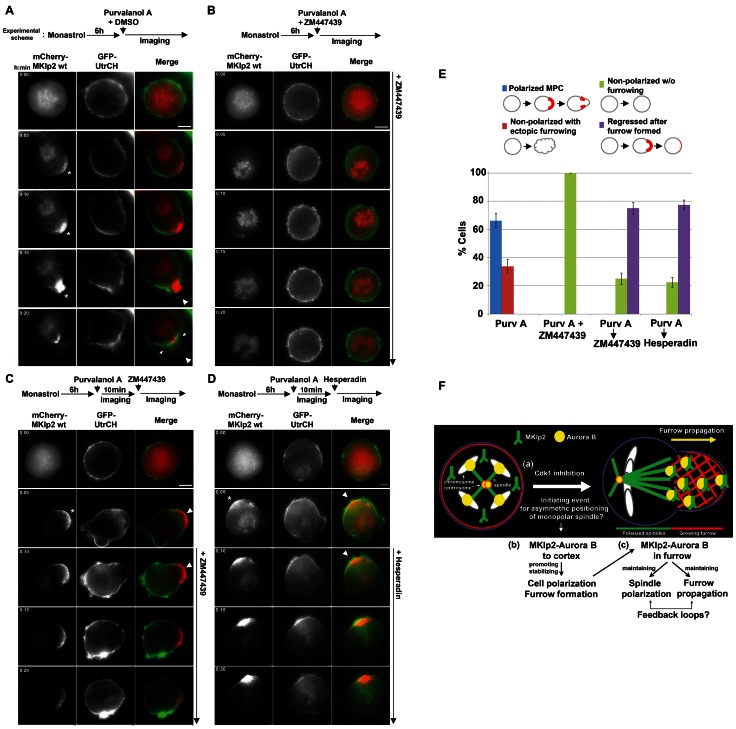
MKlp2 recruits and provides continuous Aurora B kinase activity to the growing furrow for furrow propagation and completion. (**A–D**) Time-lapse live-cell images. HeLa cells were transfected with expression vectors encoding mCherry-MKlp2 and GFP-UtrCH (cortical marker), and 24 h after transfection, the cells were subjected to monopolar cytokinesis. In panel **A**, arrows indicate where a typical polarized furrow was completed. Asterisks indicate the site where mCherry-MKlp2 accumulated at the cell cortex and the furrow. Arrow heads in panels **A**, **C** and **D** denote the sites of the growing furrow before adding ZM447439 or hesperadin. For panel **B**, Purv A and ZM447439 were added concurrently. For panels **C** and **D**, ZM447439 or hesperadin was added sequentially after purvalanol A treatment for 10 min. The fate of the same cell was continuously monitored. White bars represent 5 µm. (**E**) Monopolar cells (from **A–D**) were scored as cells that completed monopolar cytokinesis (polarized MPC), formed ectopic furrows without a polarized cortex (non-polarized with ectopic furrowing), no significant furrowing activity without a polarized cortex (non-polarized without furrowing), or formed polarized furrows but subsequently regressed (regressed after polarization). The average percentages based on three independent experiments (total *n*>100 per condition, +/− standard deviation) are shown. (**F**) Proposed model. (a) In monopolar cytokinesis, inhibition of Cdk1 triggers a symmetry-breaking reaction that initiates polarization of the monopolar spindles and the cell cortex. Although monopolar spindles are symmetrically positioned, they become asymmetric upon Cdk1 inhibition. Thus, the original event triggering the asymmetry of monopolar spindles remains unclear. (b) During this reaction, MKlp2 mediates Aurora B translocation from the monopolar spindles (or the spindle midzone) towards the cell cortex (or the equatorial cortex) in an interdependent manner. At the ends of monopolar spindles that contact the cell cortex, MKlp2 may act as the bridge between the asymmetrically polarizing monopolar spindles and the actomyosin filaments at the cell cortex. This event may stabilize the polarizing monopolar spindles towards the cell cortex. In turn, it facilitates a rapid accumulation of MKlp2-Aurora B to the cell cortex in a polarized manner. This cortical accumulation of MKlp2-Aurora B promotes furrow formation (or might constrict contractile ring in bipolar cytokinesis) via the kinase activity of Aurora B towards currently unidentified target(s). (c) Once the furrow is formed, MKlp2 targets Aurora B to actomyosin filaments in the gap region between the stably polarized monopolar spindles and the furrowing cortical cap. This event may continuously provide Aurora B kinase activity to the growing furrow for propagation and completion. Interestingly, the kinase activity of Aurora B at the growing furrow may also be essential for maintaining the monopolar spindles polarized towards the furrow (Movie S1, S2), indicating that positive feedback loops may exist that maintain polarization between the monopolar spindles and the growing furrow.

Although the kinase activity of Aurora B is required for stable furrow ingression [12], whether it functions directly at the furrow has remained elusive. MKlp2 is responsible for targeting Aurora B to the growing furrow; therefore, we tested this issue using time-lapse live-cell imaging of cells undergoing monopolar cytokinesis. First, as determined by immunofluorescence analysis, mCherry-tagged wild-type MKlp2 translocated the majority of Aurora B to the growing furrow (Figure S5B), suggesting that mCherry-MKlp2 was as functional as endogenous MKlp2. Next, using time-lapse live-cell imaging analysis, ectopically expressed mCherry-MKlp2 efficiently accumulated at the growing furrow upon Purv A treatment, and the furrow propagated and reached completion with similar kinetics compared with normal monopolar cytokinesis (Figure 6A). In contrast, when the cells were concurrently treated with Purv A and the Aurora B inhibitor ZM447439, cell polarization and furrow formation was inhibited (Figure 6B, 6E), consistent with a previous report [7]. However, this concurrent inhibition also abolished the cortical localization of mCherry-MKlp2 (Figure 6B); therefore, no cortical localization of Aurora B was observed, which suggests that MKlp2 and Aurora B are interdependently required for their relocation from anaphase chromatin to the cell cortex. This concurrent treatment also mislocalized MKlp1 from the monopolar spindles (Figure S9, panel a), creating difficulty in determining the importance of MKlp2-Aurora B at the growing furrow. Therefore, to avoid this complication, ZM447439 was sequentially added after polarizing HeLa cells with Purv A treatment (Figure 6C, 6E). Using time-lapse live-cell analysis, we first determined that mCherry-MKlp2 efficiently accumulated at the growing furrow at 10 min after Purv A treatment (Figure 6C). Then, ZM447439 was added, and the fate of the same cell was continuously monitored. Notably, upon addition of ZM447439, the furrowing activity immediately ceased, and the furrow regressed, although a considerable amount of mCherry-MKlp2 remained at the regressing furrow (Figure 6C, 6E). A similar result was also obtained using the Aurora B selective inhibitor hesperadin (Figure 6D, 6E), excluding a potential off-target effect of ZM447439. Using immunofluorescence analysis, endogenous Aurora B and MKlp2 remained at the regressing furrow under this sequential treatment condition (Figure S9, panels c & d). Also, this sequential treatment did not affect MKlp1 localization to the ends of monopolar spindles (Figure S9, panel b). Together, these results suggest that the kinase activity of Aurora B at the growing furrow is continuously required for furrow propagation and completion. Interestingly, this sequential inhibition of Aurora B also depolarized the mitotic spindle (Movie S1, S2), suggesting that the Aurora B kinase activity at the polarized cortex may be essential for maintaining the polarization of monopolar spindles. Taken together, we conclude that targeting Aurora B to the cell cortex by MKlp2 in an interdependent manner may play a role in monopolar cytokinesis by promoting polarization and efficiently maintaining the cytokinesis furrow for completion (Figure 6F). Moreover, targeting Aurora B to the equatorial cortex by MKlp2 is required for the efficient maintenance of the ingressing furrow in a partially redundant manner with MKlp1 during bipolar cytokinesis.

## Discussion

In this study, we propose that MKlp2 is an essential factor for cytokinesis that links Aurora B (and likely as the CPC) to the equatorial cortex (or the cell cortex and the growing furrow in monopolar cytokinesis). We also show that this event may contribute to the efficient maintenance of the ingressing furrow, which is necessary for successful cytokinesis. We also demonstrate that MKlp2 requires myosin-II for its localization to the equatorial cortex, and this event is necessary for recruiting Aurora B to the equatorial cortex in mammalian cells.

Although Aurora B is important for furrow ingression and completion, the role of Aurora B in promoting cytokinesis at the equatorial cortex was unclear. However, one of the major difficulties in resolving this issue involves differentiating Aurora B at the equatorial cortex from Aurora B located at the spindle midzone in mammalian cells. In this sense, we isolated a MKlp2 mutant selectively defective in localizing to the equatorial cortex, and therefore defective in targeting Aurora B and INCENP to the equatorial cortex and not the spindle midzone. Moreover, using monopolar cytokinesis, we visualized that MKlp2 localized to the ends of polarized monopolar spindles and extended to the polarized cortical actomyosin filaments in a myosin-II-dependent manner. This phenomenon may have occurred because no polarized structure formed in myosin-II depleted cells; therefore, MKlp2 was not able to localize to the cell cortex. However, unlike siRNA-mediated depletion of myosin-II, MKlp2 mutants defective in myosin-II binding were also unable to localize to the cell cortex and the growing furrow, although this mutant efficiently localized to the ends of the monopolar spindles both in polarized and non-polarized cells. Thus, our data strongly suggest that MKlp2 localizes to the cell cortex in a myosin-II-dependent manner. In turn, MKlp2 recruits Aurora B and INCENP (thus, likely as the CPC) to the cell cortex (or the equatorial cortex). Interestingly, MKlp2 localized to chromosomes upon inhibition of Cdk1 activity, and depletion of MKlp2 retained Aurora B and INCENP on the chromosomes; therefore, we speculate that the initial interaction between MKlp2 and Aurora B (or via other components of the CPC) might occur on chromosomes (and potentially in the cytoplasm), which warrants further investigation.

Importantly, this MKlp2 mutant was also unable to efficiently promote cell polarization and furrow formation during monopolar cytokinesis. This mutant also failed to maintain stable ingression of the cleavage furrow during bipolar cytokinesis, although its abilities to target Aurora B to the monopolar spindles and the spindle midzone were intact. Together, our results reveal an essential role of a MKlp2-Aurora B complex at the equatorial cortex (or the cell cortex and the growing furrow in monopolar cytokinesis) for cytokinesis, and we propose that the key role of MKlp2 in cytokinesis involves connecting Aurora B at the midzone spindles with the equatorial cortex. We also show here that kinase activity of Aurora B should be present persistently at the furrow for propagation and completion. Given that myosin-II activity is essential for furrow formation and progression, it is likely that kinase activity of Aurora B continuously targets a cortical factor promoting myosin-II activity at the growing furrow, which warrants further investigation.

In this study, we also show that MKlp2 is required for efficient maintenance of the ingressing furrow in mammalian cells. Furthermore, in the absence of MKlp1, MKlp2 becomes essential for initiating furrow ingression during bipolar cytokinesis, demonstrating the role of MKlp2 at an early stage of cytokinesis where it may function in partially redundant pathways with MKlp1. Similar to MKlp1 and MKlp2 co-depletion described in the present work, the combined effect of Plk1 and Aurora B inhibition on furrow ingression is much greater than the effected caused by Plk1 or Aurora B inhibition alone [13]. Thus, the attenuated effects of MKlp2 knockdown alone are likely a result of redundant mechanisms involved in furrow ingression during bipolar cytokinesis. Nevertheless, the cortical localization of MKlp2 becomes essential for furrow ingression in the absence of MKlp1; therefore, we favor a model that MKlp2 targets Aurora B to the equatorial cortex in order to support furrow ingression in a parallel pathway with centralspindlin during bipolar cytokinesis. Notably, the total levels of RhoA at the equatorial cortex did not change significantly in MKlp2-depleted cells, although the RhoA zone measurably expanded. Thus, our result supports the idea that MKlp2 functions in promoting highly polarized accumulation of active RhoA to the equatorial cortex but not by activating RhoA. We speculate that the failure in completing the ingressing furrow in MKlp2-depleted cells may be due to incomplete constriction of the contractile ring or cleavage furrow instability (e.g., cortical oscillation) as also observed in cells depleted of anillin, the assembly scaffold for contractile ring components [14]. Interestingly, co-depletion of anillin and MKlp1 also leads to a complete block of furrow ingression as observed in MKlp1 and MKlp2 co-depleted cells [14]. Thus, we further speculate that centralspindlin is required for RhoA activation and myosin-II accumulation at the equatorial cortex; however, MKlp2-Aurora B is recruited to the equatorial cortex through myosin-II, and it may function in focusing (or tightly organizing) the furrowing machinery. This model also supports the recently suggested role of Aurora B in remodeling the contractile ring in *Caenorhabditis elegans* [15]. However, MKlp2 is evolutionary well-conserved from humans to *Xenopus laevis*, but it is poorly conserved in *Drosophila melanogaster* and not found in *Caenorhabditis elegans*. Thus, the essential role of MKlp2-Aurora B in cytokinesis has apparently evolved specifically in higher organisms, and their different requirements in cytokinesis among species are interesting topics for future study.

Unlike bipolar cytokinesis, we also showed that MKlp2 alone is essential for stable polarization and furrow formation in monopolar cytokinesis, specifically by targeting Aurora B to the cell cortex. Initial events for stable polarization and breaking the symmetry of monopolar spindles are required for monopolar cytokinesis, whereas the symmetry is already broken in bipolar cytokinesis by positioning the mitotic spindles relative to the chromosomes aligned in the metaphase plate. Thus, although the original event triggering the asymmetry of monopolar spindles is unclear, we propose that MKlp2-Aurora B may be important for symmetry breaking in monopolar cytokinesis, and we hypothesize that MKlp2-Aurora B may also contribute to these aspects in bipolar cytokinesis.

Positive feedback between the cell cortex and the mitotic spindles involving Aurora B has been suggested to stabilize polarized microtubule towards the cell cortex [7]. This notion is consistent with our observations involving monopolar cytokinesis that suggest that (i) MKlp2 localizes between the polarizing monopolar spindles and the cortical actomyosin filaments, which may stabilize the monopolar spindles toward the cell cortex in a polarized manner, (ii) MKlp2 recruits Aurora B in an interdependent manner to actomyosin filaments at the growing furrow to provide persistent Aurora B activity for furrow propagation and completion, and (iii) this Aurora B activity is also required for maintaining the monopolar spindles polarized towards the growing furrow, while inhibiting its kinase activity depolarized the monopolar spindles (Figure 6C–E). However, monopolar spindles are symmetrically positioned, yet they become asymmetric upon Cdk1 inhibition; therefore, the original event triggering the asymmetry of monopolar spindles remains unknown. Nevertheless, our data presented here support the idea that MKlp2 may mediate the proposed positive feedback loops that promotes microtubule polarization and furrowing by connecting and recruiting Aurora B from the polarizing mitotic spindles to the cell cortex, and the initiating event of symmetry breaking warrants further investigation.

## Materials and Methods

### Cell Lines, Culture and Reagents

HeLa cells, including the cells stably expressing GFP-α-tubulin and GFP-Histone H2B, were maintained in DMEM supplemented with 10% fetal bovine serum (Invitrogen). Vectors encoding HA-MKlp2 and MKlp1 were previously described [16]. Vectors encoding mCherry-MKlp2 were constructed by inserting PCR-derived mCherry cDNA into pCAN1-HA-MKlp2. MKlp2-deletion mutants were generated by PCR with appropriate primers and cloned into pCAN1-HA. The GFP-UtrCH [17] and GFP-myosin-II constructs [18] were previously described. For the Dox-inducible system, MKlp2 cDNA was cloned into pRetroX-Tight-puro-3xFlag. Retroviral infection was produced using the PLAT-A packaging cell line and the HeLa Tet-On Advanced Cell Line for infection (Clontech). Cell lines were selected with puromycin (0.5 µg/ml) and maintained in DMEM with 10% Tet-system-approved FBS (Clontech). To induce Flag-MKlp2 expression, the cells were treated with doxycycline (5 µg/ml; Sigma). Control non-silencing (Qiagen) or specific siRNA against MKlp2 (5′-AACGAACTGCTTTATGACCTA-3′) and MKlp1 (5′-CAGAAGTTGAAGTGAAATCTA-3′) were used. For Figure 4B, HeLa cells were co-transfected with 50 nM each of siRNAs specific for myosin-II-A (SI02654911) and myosin-II-B (SI02653672; Qiagen).

### Cell Synchronization and Monopolar Cytokinesis

For time-lapse live-cell analysis (Figure 1A, 3D), the cells were synchronized at the G_1_/S boundary by exposure to 2 mM thymidine for 16 h and incubated in fresh medium for 10 h. During this time, the cells were transfected with siRNAs (100 nM) with Lipofectamine 2000 (Invitrogen) and re-exposed to 2 mM thymidine for 14 h. For Figure 3D, doxycycline (5 µg/ml) was added to the second thymidine block. For monopolar cytokinesis analysis, HeLa cells were treated with the Eg5 inhibitor monastrol (100 µM; Santa Cruz Biotechnology) for 6 h and with the Cdk1 inhibitor purvalanol A (30 µM; Sigma) at 37°C for 15 min (Figure 4A-C) or 20 min (Figure 5A–C) before fixation with ice-cold methanol for 3 min. For Figure 6B–D, Aurora B inhibitor was added [ZM447439 at 2 µM (BIOMOL International); hesperadin at 200 nM (Selleck Chemicals)] either concurrently or 10 min after purvalanol A treatment.

### Immunofluorescence and Time-lapse Live-cell Imaging

HeLa cells grown on coverglass-bottom chamber slides (Lab Tek) were fixed with ice-cold methanol for 3 min. For cortical localization of RhoA (Figure 1D), 10% trichloroacetic acid (TCA) in phosphate-buffered saline (PBS) was used for fixation for 10 min on ice. The fixed cells were permeabilized with 0.5% Triton X-100 and exposed to PBS containing 4% BSA. Primary antibodies diluted in PBS containing 1% BSA and 0.1% Triton X-100 including antibodies specific to MKlp2 (B01, Abnova; A300-879A, Bethyl Laboratories; 1∶200 dilution), myosin-II (ab24762, Abcam; 1∶200 dilution), Flag (PM020, 22381, MBL; 1∶200 dilution), Aurora B (13E8A7; 1∶100 dilution), INCENP (H153; 1∶100 dilution), RhoA (119, 26C4; 1∶100 dilution), MKlp1 (N19) and PRC1 (H70, Santa Cruz Biotechnology; 1∶100 dilution), RacGAP1 (generous gift from T. Kitamura, Tokyo University; 1∶1,000 dilution), and tubulin (AA13, Sigma; ab6046, Abcam; 1∶500 dilution) as well as isotype-specific secondary antibodies (1∶500 dilution) coupled to Alexa Fluor 488, 594, or Cy5 (Molecular Probes) were used. Cells were counterstained with Hoechst 33342 (Thermo Scientific) at 1 µg/ml. Images were acquired at RT with the 3D-SIM mode using a Super Resolution Microscope (Nikon) equipped with an iXon ^EM^+885 EMCCD camera (Andor) mounted on a Nikon Eclipse Ti-E inverted microscope with a CFI Apo TIRF (100x/1.40 oil) objective and processed using NIS-Elements AR software. For time-lapse live-cell analysis (Figure 4E, Figure 6A–D), a Stage Top Incubation with Digital CO_2_ mixer (Tokai) was used, and images were acquired at 37°C using the 2D-SIM mode. For Figure 3A and 3C, images were acquired at 25°C with an AxioCam HRc (Carl Zeiss) camera mounted on an Axiovert microscope (Axiovert 100M, Carl Zeiss) with a Plan-Aphochromat (100x/1.40 oil) objective. Images were deconvoluted with LSM 5 image software.

### Immunoblotting and Immunoprecipitation

Protein was prepared with 1% NP-40 cell lysis buffer [50 mM Tris HCl (pH 8.0), 120 mM NaCl, 1% NP-40] containing 1 mM DTT, protease inhibitor mix (Complete Mini, Roche) and phosphatase inhibitor cocktails 2 and 3 (Sigma), and 20 µg of protein was loaded for SDS-PAGE. The primary antibodies (1∶1,000 dilution) used included MKlp2 (A300-879A), myosin-II (A302-042A, Bethyl Laboratories), RhoA (26C4), HA (Y-11), GFP (FL, Santa Cruz Biotechnology), and β-actin (Sigma), and the secondary antibodies used were sheep anti-mouse IgG HRP and donkey anti-rabbit IgG HRP (Amersham; 1∶2,000 dilution). Immunoreactive proteins were visualized using ECL reagent (Amersham). For Figure 2A–D, cells were lysed in 0.1% NP-40 cell lysis buffer and subjected to immunoprecipitation with 2 µg of antibodies against HA (12CA5), Myc (9E10) and GFP (FL, Santa Cruz Biotechnology) with Protein G-Sepharose beads (Sigma), or myosin-II (A302-042A, Bethyl Laboratories) and MKlp2 (A300-878A, Bethyl laboratory) with Protein A-Sepharose beads (Sigma). The beads were washed with 0.1% NP-40 cell lysis buffer and subjected to immunoblot analysis.

### Actin Co-sedimentation Assays

For the actin co-sedimentation assay (Figure 2F), HeLa cells were grown in 6-well plates and transfected with 4 µg of HA-MKlp2 constructs. Next, 24 h after transfection, the cells were harvested in 0.1% NP-40 cell lysis buffer. Nocodazole (200 ng/ml) was added to the cell lysates to disrupt polymerized microtubules. Overall, 100 µg of total cell lysates were subjected to the actin co-sedimentation assay using an actin binding spin-down assay kit containing non-muscle actin (BK013, Cytoskeleton).

### Protein Expression, Purification and GST-pulldown Analysis

For recombinant proteins, pGEX-6P-1 encoding MKlp2 was transformed into BL21(DE3) competent cells and induced with 0.1 mM IPTG overnight at 16°C. Cells were lysed by sonication in Buffer A [50 mM Tris-HCl (pH 8.5), 200 mM NaCl, 3 mM DTT, 5% Glycerol, 1 mM PMSF]. Proteins were bound to glutathione-sepharose beads (GE Healthcare) for 2 hours at 4°C, washed 3 times with Buffer A, and eluted with reduced glutathione (10 mM). Eluted proteins were further purified using Superdex 200 column equilibrated with Buffer B [80 mM PIPES (pH 7.0), 2 mM MgCl_2_, 0.5 mM EGTA). For *in vitro* translation and GST pull-down analysis, Myc-myosin-II proteins were translated *in vitro* in the presence of a mixture of [^35^S]cysteine and [^35^S]methionine (Perkin-Elmer) with the use of the TNT® Coupled reticulocyte lysate system (Promega). GST-tagged MKlp2 was loaded onto glutathione-agarose beads for 30 min at 4°C in the presence of NP-40 cell lysis buffer [50 mM Tris-HCl (pH 8.0), 120 mM NaCl, 1% NP-40] containing 1 mM DTT, protease inhibitor mix (Complete Mini, Roche), and phosphatase inhibitor mix I and II (Sigma). The beads were washed with the cell lysis buffer and then incubated overnight at 4°C with *in vitro*-translated proteins. The beads were then isolated and washed before the addition of SDS sample buffer.

## Supporting Information

Figure S1 **MKlp2 is required for the highly focused accumulation of RhoA at the equatorial cortex.** (**A**) MKlp2 contributes to the highly focused accumulation of RhoA at the equatorial cortex. Immunofluorescence analysis was performed at 30 h after transfection with the indicated siRNAs. Representative images of maximum intensity projections of serial optical sections through the equatorial cortex are presented (from 15 sections with 0.4 µm of thickness) to show a less focused RhoA zone at the equatorial cortex in MKlp2-depleted cells (panels c, d) compared with control cells (panels a, b). Insets represent the boxed area. Cells in early (panels a, c) and late anaphase (panels b, d) are shown. The average lengths of the RhoA zone at the equatorial cortex (*n* = 20 cells per each bar) of early anaphase cells are shown with error bars (right graph). (**B**) Depleting MKlp2 does not significantly affect the levels of centralspindlin or the CPC. HeLa cells were transfected with control or various different siRNAs against MKlp2 (50 nM; the siRNA sequences are shown in bottom panel). At 24 h after transfection, the cells were treated with nocodazole (200 ng/ml) for an additional 6 h before harvesting to increase the mitotic population of cells. Immunoblot analysis using 20 µg of total cell lysates was performed using the indicated antibodies. Relative band intensities to control siRNA are shown. (**C**) Generation of the Dox-inducible Flag-MKlp2 system. Structural motifs of MKlp2 with siRNA target sequences that were mutated to generate expression vectors resistant to MKlp2 siRNA (top). After the HeLa cells were transfected with the indicated siRNAs, the cells were treated with Dox (5 µg/ml), and after 30 h, they were lysed and then subjected to immunoblot analysis (bottom panel). Note that both the monoclonal and polyclonal antibodies used in this study to detect endogenous MKlp2 were raised against the C-terminal domain of MKlp2. Thus, both antibodies were able to detect endogenous MKlp2 and wild-type Flag-MKlp2(1-890), and they were unable to detect Dox-induced Flag-MKlp2(1-842) (top panel). However, using the antibodies raised against the Flag-epitope, it was confirmed that Flag-MKlp2(1-842) was expressed at comparable levels to Flag-MKlp2(1-890) (middle panel) and that the expression levels of Flag-MKlp2(1-842) were not affected by transfection with MKlp2 siRNA #3, while the levels of endogenous MKlp2 were markedly decreased (lanes 5–8). (**D**) Dox-induced Flag-MKlp2 (treated as in panel **C**) rescues RhoA focusing at the equatorial cortex. Immunofluorescence analysis of cells in early anaphase. For panels **A** and **D**, the cells were fixed with 10% trichloroacetic acid (TCA) in PBS to visualize RhoA at the equatorial cortex and stained with the indicated antibodies. All images were acquired using 3D-SIM analysis. White bars represent 5 µm.
(TIF)

Figure S2 **MKlp2(1-842) binds to microtubules, Aurora B and Plk1 as comparable to MKlp2(1-890).** (**A, B**) Lysates of HeLa cells expressing the indicated Flag-MKlp2 (A) or purified recombinant GST-MKlp2 (**B**) were supplemented with taxol-stabilized microtubules (except lanes 1-4) and subjected to ultracentrifugation at 40,000 rpm for 40 min at RT. The resulting supernatant (S) and pellet (P) fractions were then subjected to immunoblot analysis with the indicated antibodies (A) or silver staining (**B**). (**C**) mCherry-MKlp2(1-842) was transiently expressed in HeLa cells stably expressing GFP-α-tubulin, and the microtubule localization of mCherry-MKlp2(1-842) was determined using 3D-SIM analysis. Bar, 5 µm. Note that the majority of mCherry-MKlp2(1-842) co-localized with GFP-α-tubulin. (**D**) HeLa cell lysates expressing the indicated HA-MKlp2 with Flag-Aurora B or Flag-Plk1 were subjected to immunoprecipitation with antibodies against HA. The precipitates were analyzed by immunoblot assay using the indicated antibodies.
(TIF)

Figure S3 **Centralspindlin properly localizes to the spindle midzone in MKlp2-depleted HeLa cells expressing Dox-induced Flag-MKlp2(1-890) and Flag-MKlp2(1-842).** (**A**) Immunoblot analysis of stable HeLa cell lines inducibly expressing Flag-MKlp2 after treatment with the indicated amounts of doxycyclin (Dox) for 30 h. Arrows indicate Dox-induced Flag-MKlp2 and endogenous MKlp2 expression. (**B**) Co-localization of Dox (5 µg/ml)-induced Flag-MKlp2(1-890) with Aurora B in MKlp2-depleted HeLa cells determined by immunofluorescence analysis (top panel). The cells were fixed with ice-cold methanol and stained with the indicated antibodies. A cross-section at the equator (white arrow, top panel) with an intensity profile of the corresponding section is shown (bottom graph). (**C**, **D**) Centralspindlin properly localizes to the spindle midzone in HeLa cells with Dox-induced Flag-MKlp2(1-890) and Flag-MKlp2(1-842). HeLa cells were transfected with MKlp2 siRNAs (50 nM) to deplete endogenous MKlp2, and they were treated with Dox (5 µg/ml) to express the indicated siRNA-resistant Flag-MKlp2; 30 h later, the cells were fixed with ice-cold methanol. All images were acquired using 3D-SIM analysis. Arrows indicate the equatorial cortex. Insets represent the boxed areas. White bars represent 5 µm.
(TIF)

Figure S4 **Depletion of MKlp2 does not affect the protein levels of myosin-II and MKlp1 or the tip of monopolar spindles contacting the cell cortex.** (**A, B**) Immunoblot analysis of HeLa cell lysates (from Figure 4B and 4C). Overall, 20 µg of total cell lysates was used for immunoblot analysis using the indicated antibodies. Note that depletion of myosin-II did not change the levels of MKlp2 (panel A), but it disrupted the cortical localization of MKlp2 (Figure 4B, panel b). Additionally, depletion of MKlp2 did not measurably change the levels of myosin-II and MKlp1 (panel B). Relative band intensities to control siRNA are shown. (**C**) Immunofluorescence analysis of HeLa cells stably expressing GFP-EB1 in monopolar cytokinesis. Overall, 24 h after transfection with indicated siRNAs, the cells were subjected to monopolar cytokinesis by treatment with the Eg5 inhibitor Monastrol (100 µM; Santa Cruz Biotechnology) for 6 h and with the Cdk1 inhibitor purvalanol A (30 µM; Sigma) at 37°C for 20 min. Note that the mitotic spindles and the cell cortex (determined by myosin-II staining) are polarized (white asterisk in merged image; myosin-II is shown in purple) in control cells but not in MKlp2-depleted cells, although the tips of the mitotic spindles (determined by GFP-EB1) were able to contact the cell cortex (panel b, inset).
(TIF)

Figure S5 **MKlp2(1-842) is selectively defective in recruiting Aurora B to the furrow but not to the end of monopolar spindles.** (**A**) Co-localization of Dox-induced Flag-MKlp2(1-890) and Flag-MKlp2(1-842) with Aurora B in HeLa cells undergoing monopolar cytokinesis were determined by immunofluorescence analysis. Purvalanol A (30 µM) were added for 20 min, and the cells were fixed with ice-cold methanol. Note that Flag-MKlp2(1-890) localized together with Aurora B to the cell cortex and to the furrow (top panels, white asterisk). In contrast, Flag-MKlp2(1-842) only co-localized with Aurora B in a punctate staining pattern, representing the end of monopolar spindles (bottom panels) as shown in Figure 5A (panels f-h). (**B**) mCherry-MKlp2 localizes to the cell cortex together with endogenous Aurora B. Co-localization of ectopically expressed mCherry-MKlp2 with endogenous Aurora B to the cell cortex in HeLa cells undergoing monopolar cytokinesis upon Purvalanol A treatment (as indicated in Figure 6A) was determined by immunofluorescence analysis. The cells were fixed with ice-cold methanol. White bars represent 5 µm.
(TIF)

Figure S6 **Localization of MKlp2 to the cell cortex is required for stable cell polarization and furrow formation in monopolar cytokinesis.** (**A, B**) Immunofluorescence analysis of Dox-inducible HeLa cells undergoing monopolar cytokinesis. Indicated Flag-MKlp2 was induced in HeLa cells transfected with MKlp2 siRNA for 20 h. Monopolar HeLa cells were fixed using ice-cold methanol at the indicated time after purvalanol A (30 µM) addition. For B, the cells were fixed in 10% TCA at 15 min after purvalanol (30 µM) treatment. (**A**) Note that Flag-MKlp2(1-890) gradually localized to the cell cortex containing myosin-II in a polarized manner and completed monopolar cytokinesis (panels a-d). In contrast, Flag-MKlp2(1-842) displayed punctated patterns of localization at the end of monopolar spindles and adjacent to the cell cortex; however, it failed to polarize the cells and to form a furrow (panels e-h). (**B**) In these cells, RhoA was also less focused (panels c, d) compared with control cells expressing Flag-MKlp2(1-890) (panels a, b). Images were acquired using 3D-SIM. White bars represent 5 µm.
(TIF)

Figure S7 **Time-lapse live-cell imaging analysis of mCherry-MKlp2 in monopolar cytokinesis.** (**A**–**C**) Time-lapse live-cell images. After transfecting siRNA against MKlp2 for 12 h, HeLa cells stably expressing GFP-α-tubulin were transfected with expression vectors encoding the indicated mCherry-MKlp2, and 6 h after transfection, the cells were treated with Monastrol for 12 h and subjected to monopolar cytokinesis. The images at 0∶00 (h:min) were initially captured before adding purvalanol A. Upon the addition of purvalanol A (30 µM), the images from the same cells were continuously captured every 5 min using the 3D-SIM mode. (**A**) mCherry-MKlp2(1-890) gradually localized to the cell cortex and to the growing furrow and completed monopolar cytokinesis. (**B**) In contrast, mCherry-MKlp2(1-842) displayed punctated localization patterns to the end of monopolar spindles and did not stably polarize the cells or form a furrow. (**C**) The fluorescence signals for mCherry-MKlp2 were specific because no bleed-through signals were observed from the GFP-α-tubulin channel, which was used as a single channel control. Insets represent the boxed areas. White bars represent 5 µm. (**D**) Monopolar cells (from **A**, **B**) expressing the indicated mCherry-MKlp2 were scored as cells with polarized mCherry-MKlp2 at the cell cortex (blue), polarized with MPC completion (red) or failed MPC without mCherry-MKlp2 localization at the cell cortex but only at the monopolar spindles (green). The average percentages based on three independent experiments (total *n*>100 per condition, +/− standard deviation) are shown.
(TIF)

Figure S8 **Furrowing in general is not a prerequisite for the cortical localization of MKlp2 and Aurora B.** Immunofluorescence analysis. HeLa cells were transfected with MKlp2 siRNAs (50 nM) to deplete endogenous MKlp2 and treated with Dox (5 µg/ml) to express the indicated Flag-MKlp2. Next, 20 h later, cells were treated with Monastrol (100 µM) for 6 h and briefly treated with myosin-II inhibitor (–)-Blebbistatin (100 µM) for 5 min to block furrowing activity, which was then followed by purvalanol A (30 µM) treatment for 15 min. Subsequently, cells were fixed in ice-cold methanol and evaluated for the cortical localization of MKlp2 using the indicated antibodies. Note that (–)-Blebbistatin did not prevent Flag-MKlp2(1-890) and Aurora B from localizing to the cell cortex (panels b, c). Furrowing activity was inhibited; therefore, MKlp2 and Aurora B localized to the cell cortex in a non-polarized manner (panels b, c). The polarization of microtubules and the cell cortex was also completely inhibited (panels b, c). In contrast, Flag-MKlp2(1-842) together with Aurora B failed to localize to the cell cortex, but they were accumulated at the end of monopolar spindles (panels d, e). Furthermore, Aurora B in MKlp2-depleted cells did not localize to the monopolar spindles or the cell cortex but retained the chromosomes (panel a), which confirms the specificity of our imaging analysis.
(TIF)

Figure S9 **Sequential inhibition of Aurora B after polarizing cells does not alter the localization of centralspindlin at the ends of monopolar spindles.** Immunofluorescence analysis. HeLa cells were subjected to monopolar cytokinesis. For panel a, purvalanol A (30 µM) and ZM447439 (2 µM) were added concurrently for 15 min. For panels b-d, ZM447439 was added sequentially after purvalanol A treatment for 10 min, and the cells were fixed 10 min after ZM447439 treatment in ice-cold methanol. Arrows (panel b) indicate MKlp1 localization at the ends of microtubules. Although the concurrent treatment of ZM447439 and purvalanol A suppressed the microtubule localization of MKlp1 (panel a) as previously shown [7], when Aurora B was sequentially inhibited after polarizing cells, MKlp1 still localized at the ends of microtubules (panel b). Moreover, Aurora B and MKlp2 largely localized to the polarized cortex. Under this condition, furrowing activity immediately ceased, and the furrows subsequently regressed (Figure 6C), suggesting that continuous Aurora B activity at the growing furrow is necessary for furrow completion.
(TIF)

Movie S1 **Time-lapse video microscopy recordings of HeLa cells stably expressing GFP-α-tubulin in monopolar cytokinesis.** The images were captured every 5 min upon purvalanol A treatment (30 µM) using 2D-SIM.
(AVI)

Movie S2 **The cells were treated as Movie S1 except included the addition of ZM447439 (2 µM) at 10 min after purvalanol A treatment (30 µM).** Purvalanol A initially induced the polarization of mitotic spindles growing toward the growing furrow. The images were captured every 5 min upon ZM447439 addition using 2D-SIM. Sequential Aurora B inhibition efficiently depolarized the monopolar spindles and inhibited furrowing activity followed by furrow regression.
(AVI)
