# Supplementary figures and images for: Targeting Aurora B to the Equatorial Cortex by MKlp2 Is Required for Cytokinesis
Source: PLoS One. 2013 Jun 4;8(6):e64826. doi: 10.1371/journal.pone.0064826 (PMC3672163; doi:10.1371/journal.pone.0064826)

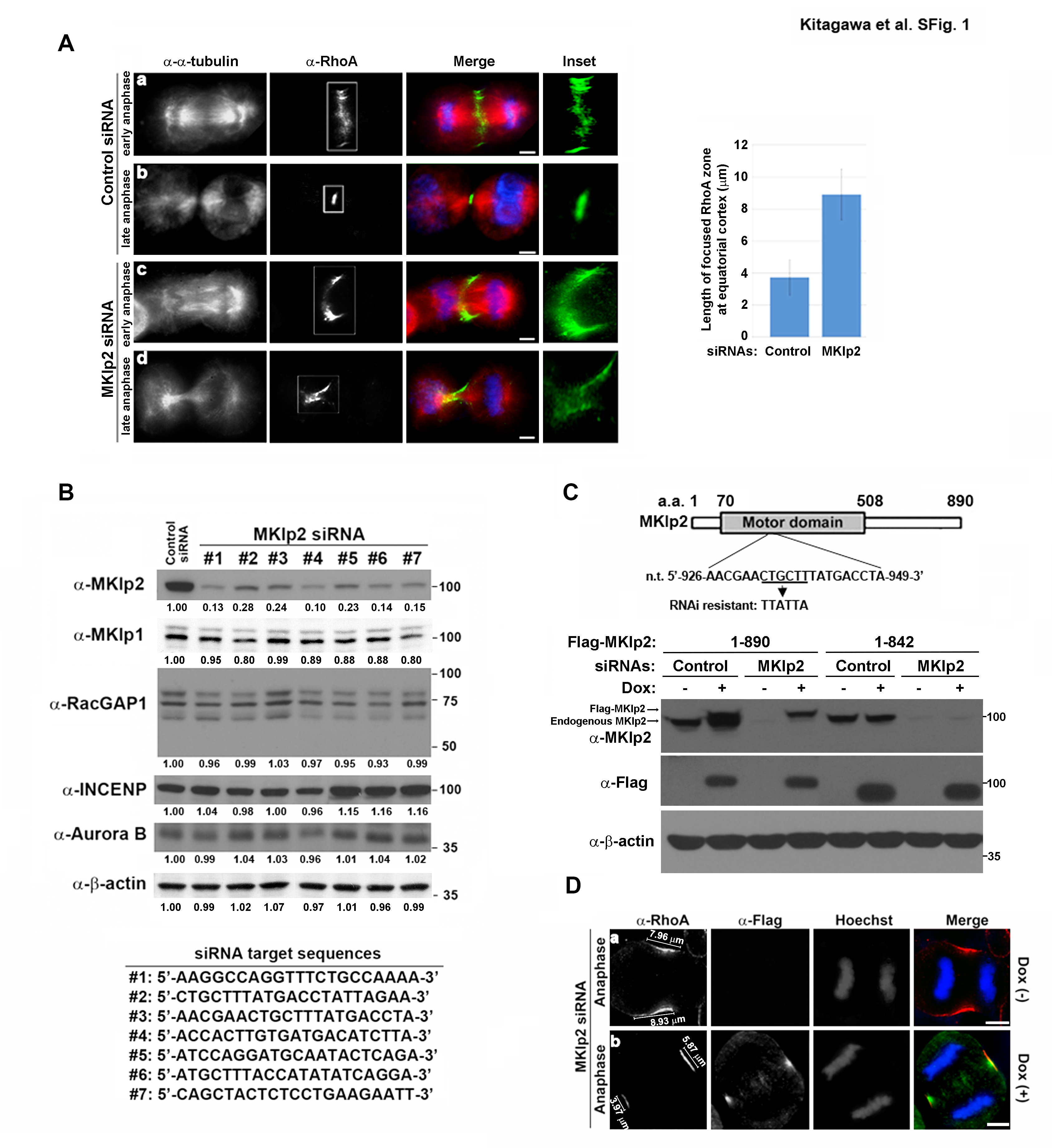

Supplement: Figure S1 — MKlp2 is required for the highly focused accumulation of RhoA at the equatorial cortex. (A) MKlp2 contributes to the highly focused accumulation of RhoA at the equatorial cortex. Immunofluorescence analysis was performed at 30 h after transfection with the indicated siRNAs. Representative images of maximum intensity projections of serial optical sections through the equatorial cortex are presented (from 15 sections with 0.4 µm of thickness) to show a less focused RhoA zone at the equatorial cortex in MKlp2-depleted cells (panels c, d) compared with control cells (panels a, b). Insets represent the boxed area. Cells in early (panels a, c) and late anaphase (panels b, d) are shown. The average lengths of the RhoA zone at the equatorial cortex (n = 20 cells per each bar) of early anaphase cells are shown with error bars (right graph). (B) Depleting MKlp2 does not significantly affect the levels of centralspindlin or the CPC. HeLa cells were transfected with control or various different siRNAs against MKlp2 (50 nM; the siRNA sequences are shown in bottom panel). At 24 h after transfection, the cells were treated with nocodazole (200 ng/ml) for an additional 6 h before harvesting to increase the mitotic population of cells. Immunoblot analysis using 20 µg of total cell lysates was performed using the indicated antibodies. Relative band intensities to control siRNA are shown. (C) Generation of the Dox-inducible Flag-MKlp2 system. Structural motifs of MKlp2 with siRNA target sequences that were mutated to generate expression vectors resistant to MKlp2 siRNA (top). After the HeLa cells were transfected with the indicated siRNAs, the cells were treated with Dox (5 µg/ml), and after 30 h, they were lysed and then subjected to immunoblot analysis (bottom panel). Note that both the monoclonal and polyclonal antibodies used in this study to detect endogenous MKlp2 were raised against the C-terminal domain of MKlp2. Thus, both antibodies were able to detect endogenous MKlp2 and w [file pone.0064826.s001.tif]

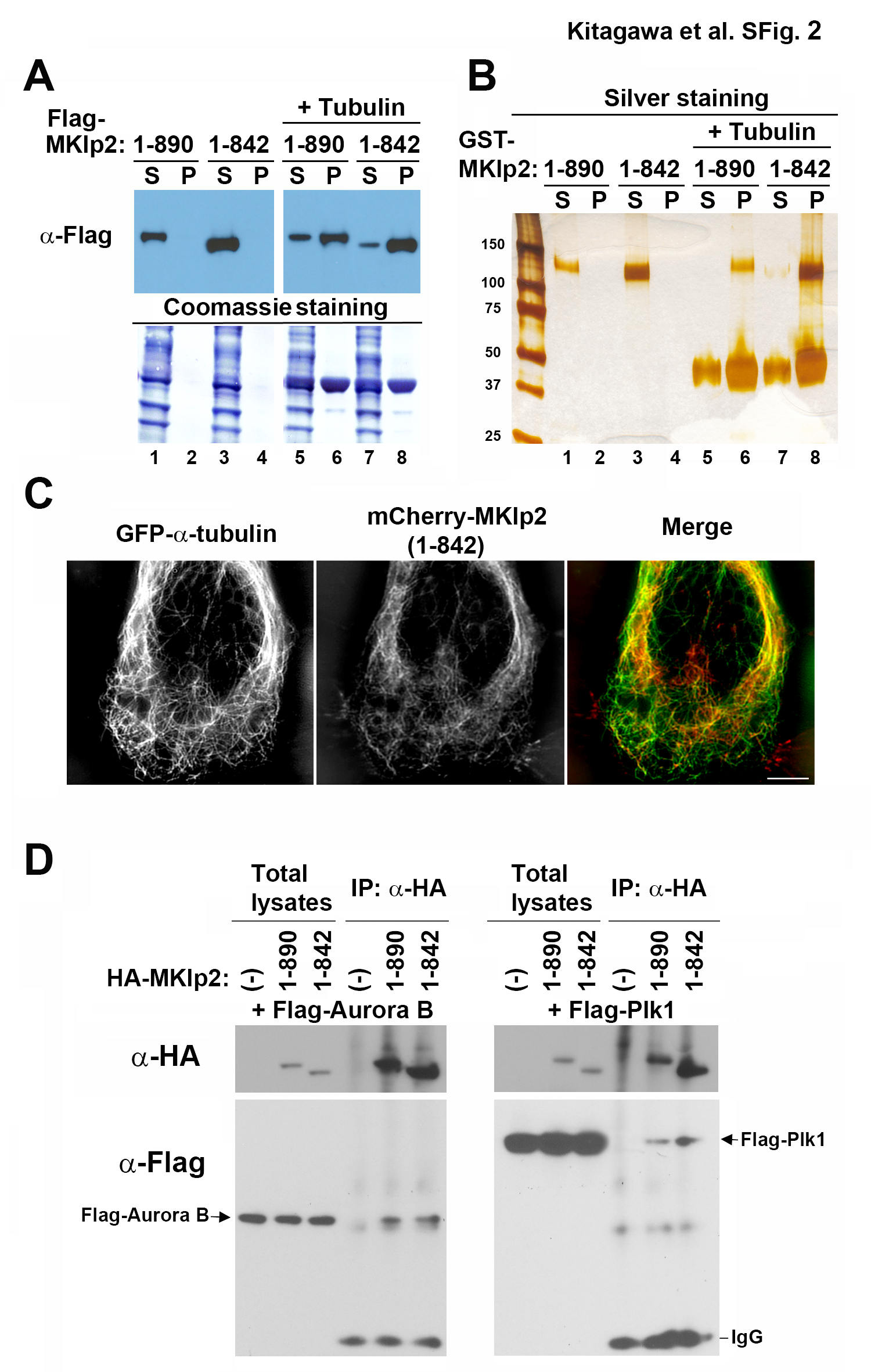

Supplement: Figure S2 — MKlp2(1-842) binds to microtubules, Aurora B and Plk1 as comparable to MKlp2(1-890). (A, B) Lysates of HeLa cells expressing the indicated Flag-MKlp2 (A) or purified recombinant GST-MKlp2 (B) were supplemented with taxol-stabilized microtubules (except lanes 1-4) and subjected to ultracentrifugation at 40,000 rpm for 40 min at RT. The resulting supernatant (S) and pellet (P) fractions were then subjected to immunoblot analysis with the indicated antibodies (A) or silver staining (B). (C) mCherry-MKlp2(1-842) was transiently expressed in HeLa cells stably expressing GFP-α-tubulin, and the microtubule localization of mCherry-MKlp2(1-842) was determined using 3D-SIM analysis. Bar, 5 µm. Note that the majority of mCherry-MKlp2(1-842) co-localized with GFP-α-tubulin. (D) HeLa cell lysates expressing the indicated HA-MKlp2 with Flag-Aurora B or Flag-Plk1 were subjected to immunoprecipitation with antibodies against HA. The precipitates were analyzed by immunoblot assay using the indicated antibodies. (TIF) [file pone.0064826.s002.tif]

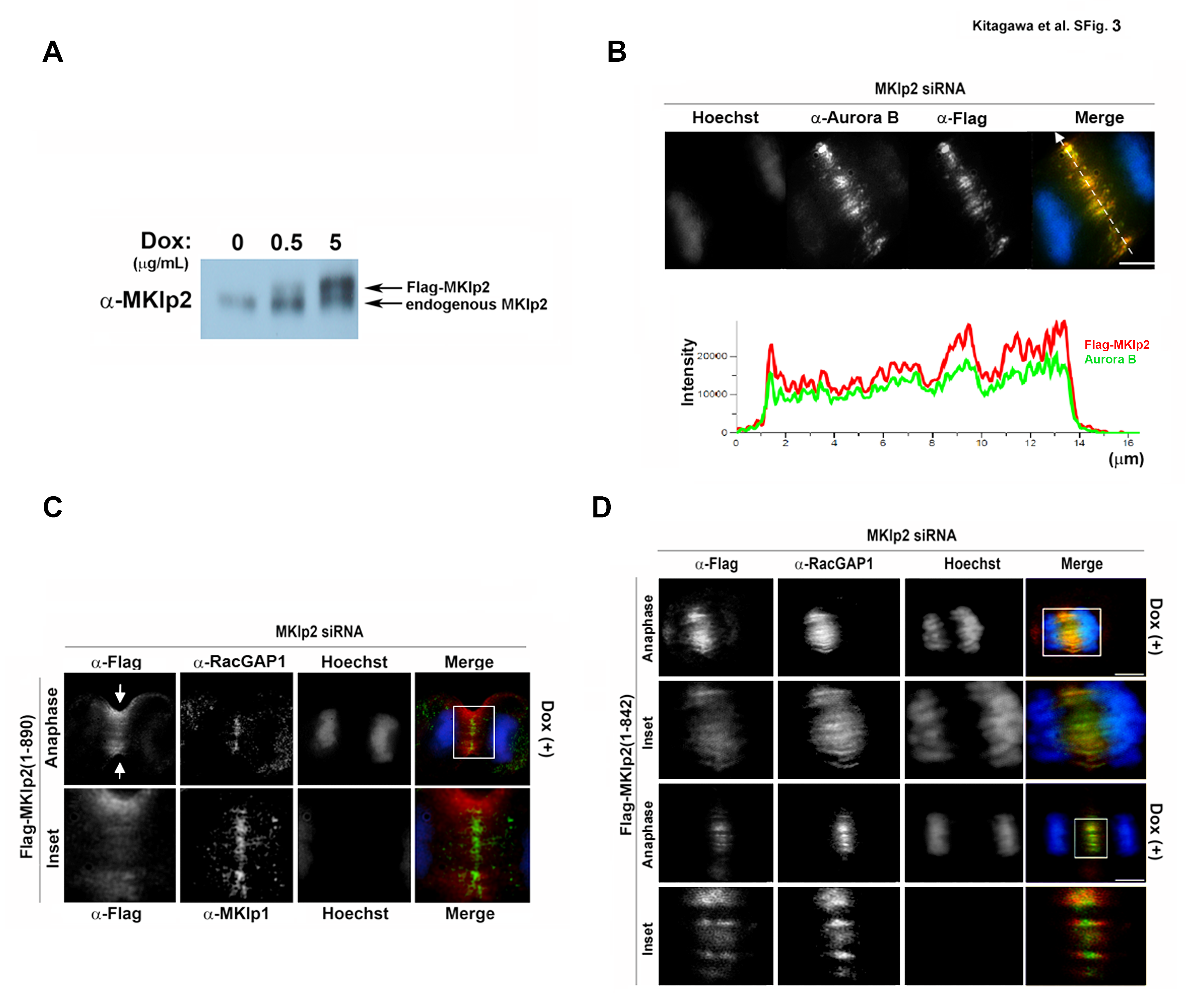

Supplement: Figure S3 — Centralspindlin properly localizes to the spindle midzone in MKlp2-depleted HeLa cells expressing Dox-induced Flag-MKlp2(1-890) and Flag-MKlp2(1-842). (A) Immunoblot analysis of stable HeLa cell lines inducibly expressing Flag-MKlp2 after treatment with the indicated amounts of doxycyclin (Dox) for 30 h. Arrows indicate Dox-induced Flag-MKlp2 and endogenous MKlp2 expression. (B) Co-localization of Dox (5 µg/ml)-induced Flag-MKlp2(1-890) with Aurora B in MKlp2-depleted HeLa cells determined by immunofluorescence analysis (top panel). The cells were fixed with ice-cold methanol and stained with the indicated antibodies. A cross-section at the equator (white arrow, top panel) with an intensity profile of the corresponding section is shown (bottom graph). (C, D) Centralspindlin properly localizes to the spindle midzone in HeLa cells with Dox-induced Flag-MKlp2(1-890) and Flag-MKlp2(1-842). HeLa cells were transfected with MKlp2 siRNAs (50 nM) to deplete endogenous MKlp2, and they were treated with Dox (5 µg/ml) to express the indicated siRNA-resistant Flag-MKlp2; 30 h later, the cells were fixed with ice-cold methanol. All images were acquired using 3D-SIM analysis. Arrows indicate the equatorial cortex. Insets represent the boxed areas. White bars represent 5 µm. (TIF) [file pone.0064826.s003.tif]

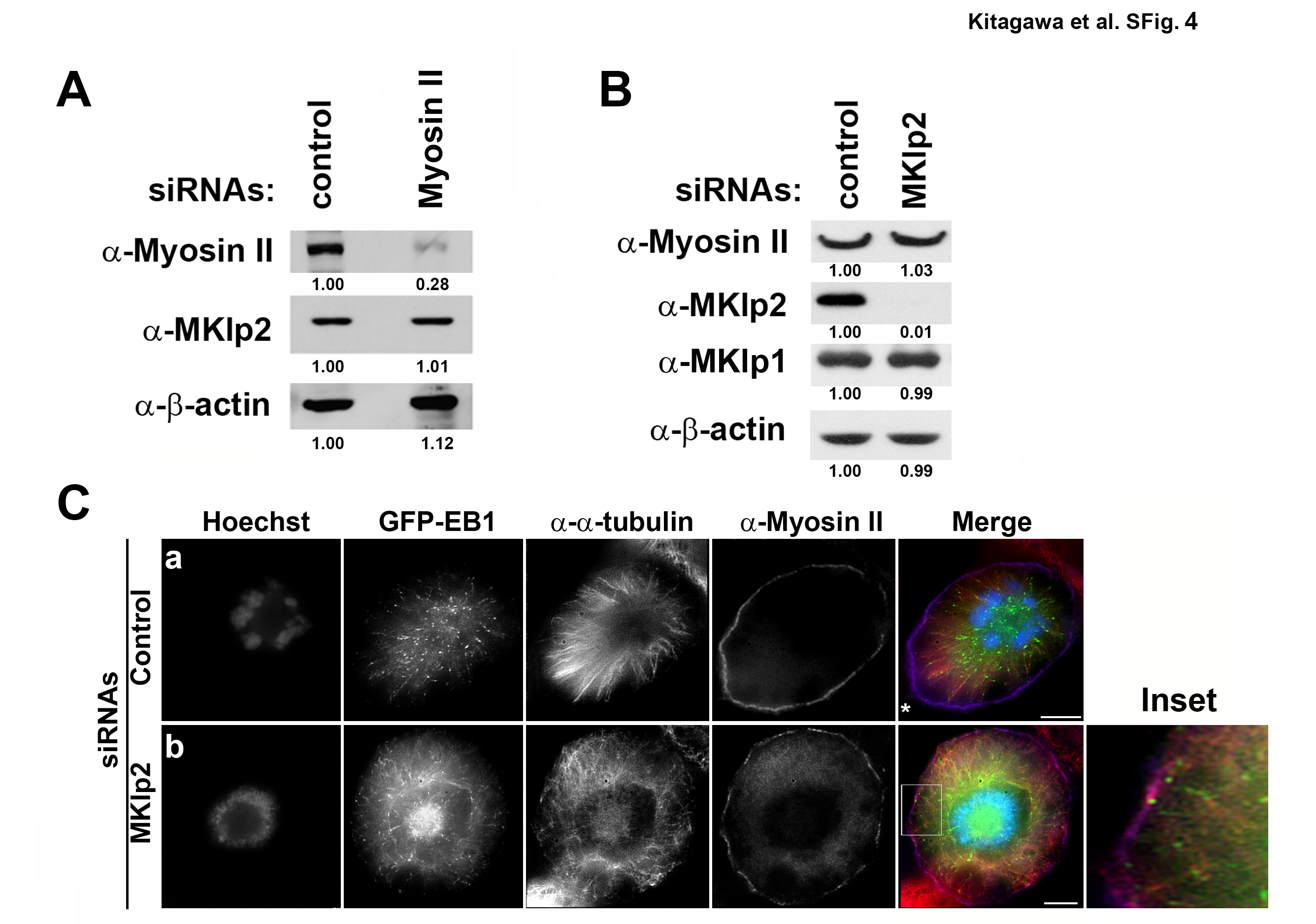

Supplement: Figure S4 — Depletion of MKlp2 does not affect the protein levels of myosin-II and MKlp1 or the tip of monopolar spindles contacting the cell cortex. (A, B) Immunoblot analysis of HeLa cell lysates (from Figure 4B and 4C). Overall, 20 µg of total cell lysates was used for immunoblot analysis using the indicated antibodies. Note that depletion of myosin-II did not change the levels of MKlp2 (panel A), but it disrupted the cortical localization of MKlp2 (Figure 4B, panel b). Additionally, depletion of MKlp2 did not measurably change the levels of myosin-II and MKlp1 (panel B). Relative band intensities to control siRNA are shown. (C) Immunofluorescence analysis of HeLa cells stably expressing GFP-EB1 in monopolar cytokinesis. Overall, 24 h after transfection with indicated siRNAs, the cells were subjected to monopolar cytokinesis by treatment with the Eg5 inhibitor Monastrol (100 µM; Santa Cruz Biotechnology) for 6 h and with the Cdk1 inhibitor purvalanol A (30 µM; Sigma) at 37°C for 20 min. Note that the mitotic spindles and the cell cortex (determined by myosin-II staining) are polarized (white asterisk in merged image; myosin-II is shown in purple) in control cells but not in MKlp2-depleted cells, although the tips of the mitotic spindles (determined by GFP-EB1) were able to contact the cell cortex (panel b, inset). (TIF) [file pone.0064826.s004.tif]

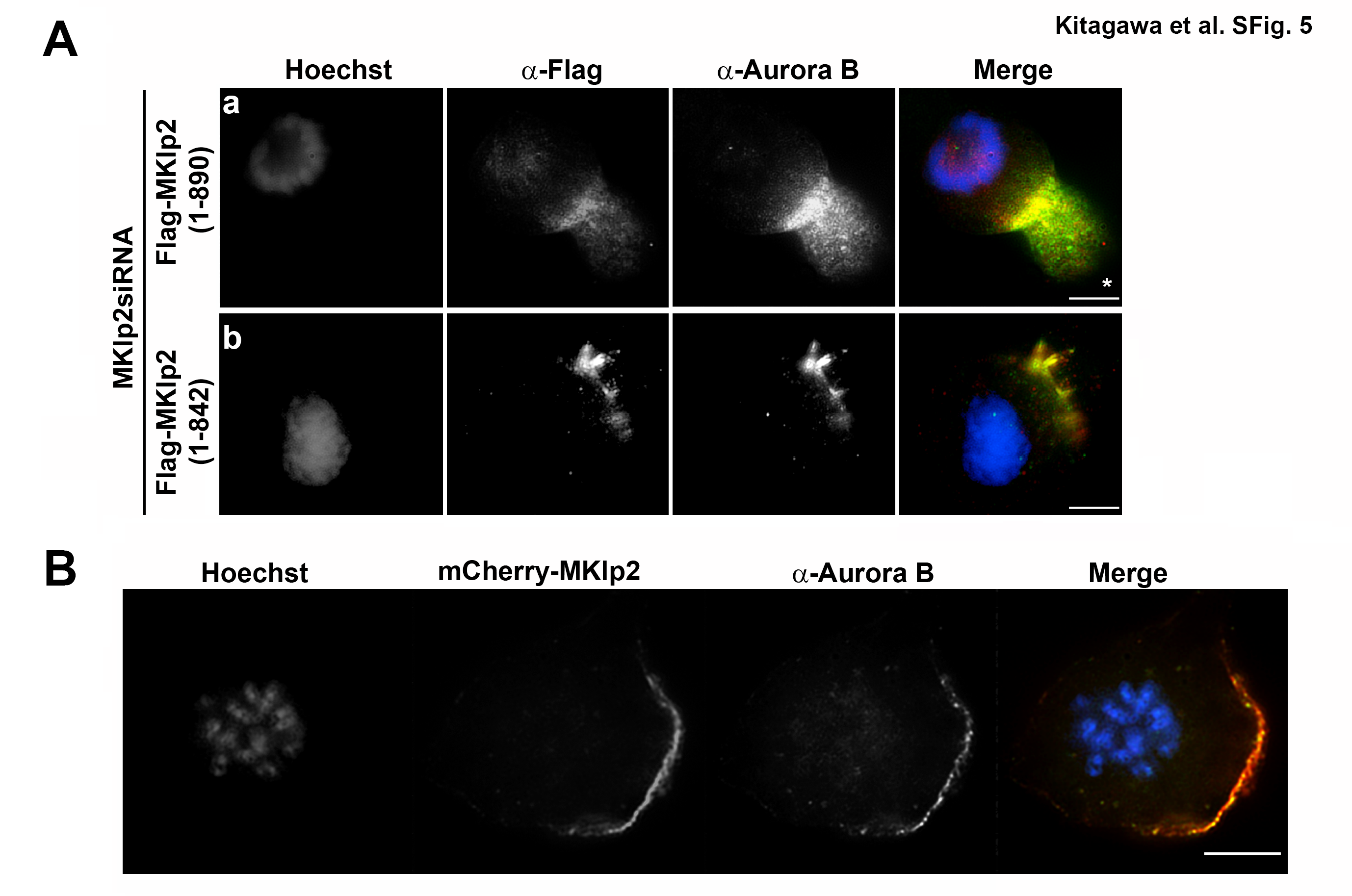

Supplement: Figure S5 — MKlp2(1-842) is selectively defective in recruiting Aurora B to the furrow but not to the end of monopolar spindles. (A) Co-localization of Dox-induced Flag-MKlp2(1-890) and Flag-MKlp2(1-842) with Aurora B in HeLa cells undergoing monopolar cytokinesis were determined by immunofluorescence analysis. Purvalanol A (30 µM) were added for 20 min, and the cells were fixed with ice-cold methanol. Note that Flag-MKlp2(1-890) localized together with Aurora B to the cell cortex and to the furrow (top panels, white asterisk). In contrast, Flag-MKlp2(1-842) only co-localized with Aurora B in a punctate staining pattern, representing the end of monopolar spindles (bottom panels) as shown in Figure 5A (panels f-h). (B) mCherry-MKlp2 localizes to the cell cortex together with endogenous Aurora B. Co-localization of ectopically expressed mCherry-MKlp2 with endogenous Aurora B to the cell cortex in HeLa cells undergoing monopolar cytokinesis upon Purvalanol A treatment (as indicated in Figure 6A) was determined by immunofluorescence analysis. The cells were fixed with ice-cold methanol. White bars represent 5 µm. (TIF) [file pone.0064826.s005.tif]

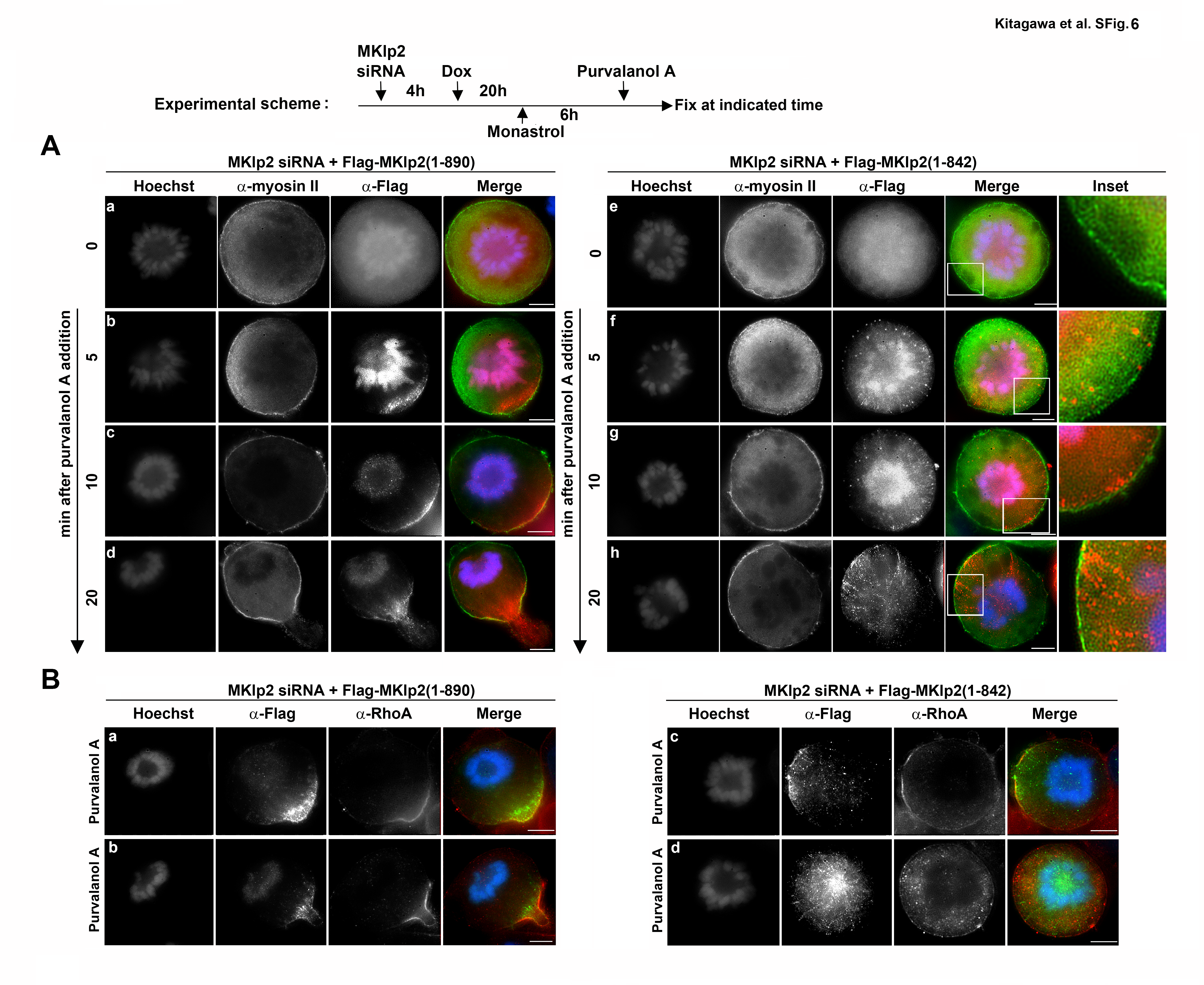

Supplement: Figure S6 — Localization of MKlp2 to the cell cortex is required for stable cell polarization and furrow formation in monopolar cytokinesis. (A, B) Immunofluorescence analysis of Dox-inducible HeLa cells undergoing monopolar cytokinesis. Indicated Flag-MKlp2 was induced in HeLa cells transfected with MKlp2 siRNA for 20 h. Monopolar HeLa cells were fixed using ice-cold methanol at the indicated time after purvalanol A (30 µM) addition. For B, the cells were fixed in 10% TCA at 15 min after purvalanol (30 µM) treatment. (A) Note that Flag-MKlp2(1-890) gradually localized to the cell cortex containing myosin-II in a polarized manner and completed monopolar cytokinesis (panels a-d). In contrast, Flag-MKlp2(1-842) displayed punctated patterns of localization at the end of monopolar spindles and adjacent to the cell cortex; however, it failed to polarize the cells and to form a furrow (panels e-h). (B) In these cells, RhoA was also less focused (panels c, d) compared with control cells expressing Flag-MKlp2(1-890) (panels a, b). Images were acquired using 3D-SIM. White bars represent 5 µm. (TIF) [file pone.0064826.s006.tif]

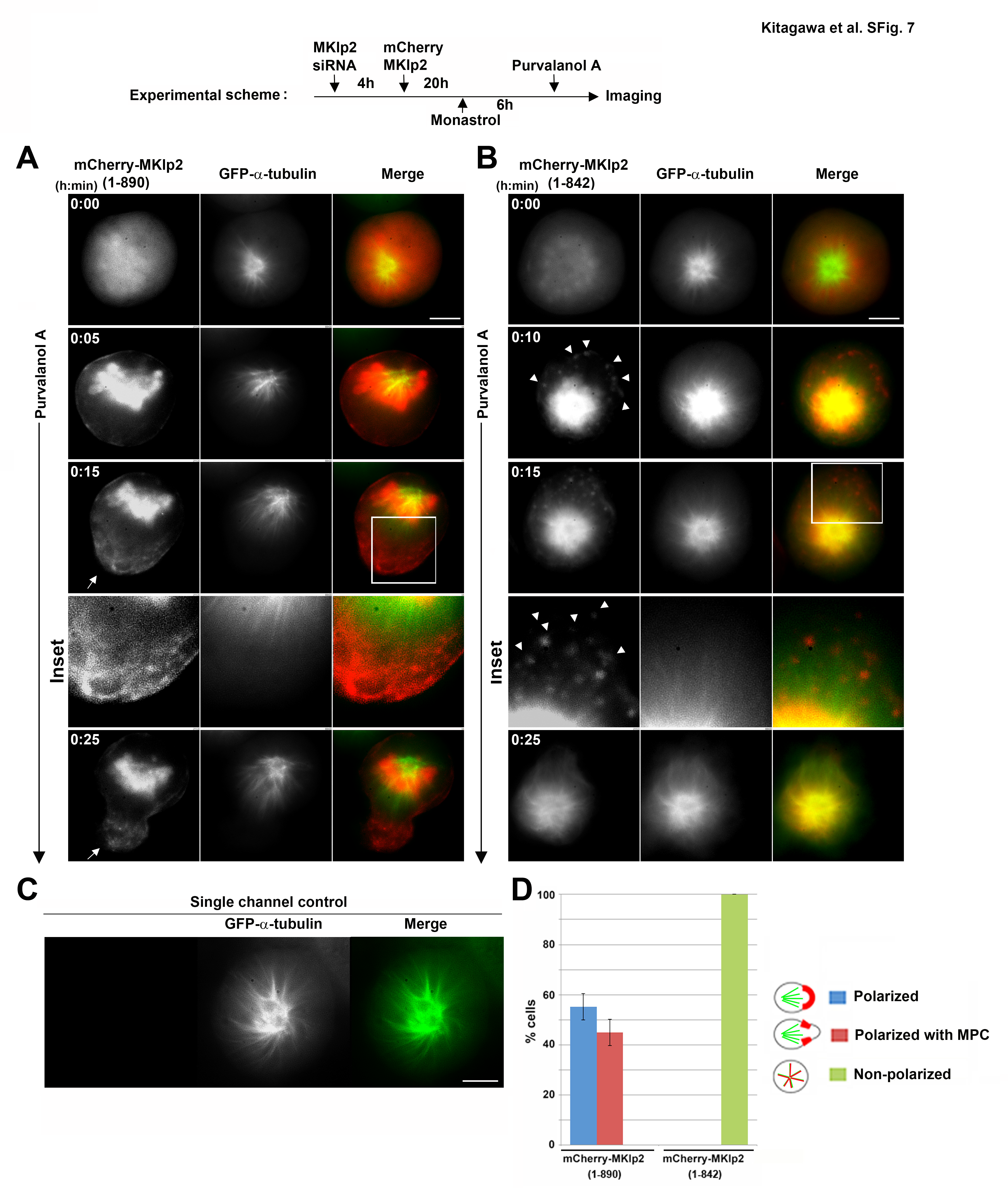

Supplement: Figure S7 — Time-lapse live-cell imaging analysis of mCherry-MKlp2 in monopolar cytokinesis. (A–C) Time-lapse live-cell images. After transfecting siRNA against MKlp2 for 12 h, HeLa cells stably expressing GFP-α-tubulin were transfected with expression vectors encoding the indicated mCherry-MKlp2, and 6 h after transfection, the cells were treated with Monastrol for 12 h and subjected to monopolar cytokinesis. The images at 0∶00 (h:min) were initially captured before adding purvalanol A. Upon the addition of purvalanol A (30 µM), the images from the same cells were continuously captured every 5 min using the 3D-SIM mode. (A) mCherry-MKlp2(1-890) gradually localized to the cell cortex and to the growing furrow and completed monopolar cytokinesis. (B) In contrast, mCherry-MKlp2(1-842) displayed punctated localization patterns to the end of monopolar spindles and did not stably polarize the cells or form a furrow. (C) The fluorescence signals for mCherry-MKlp2 were specific because no bleed-through signals were observed from the GFP-α-tubulin channel, which was used as a single channel control. Insets represent the boxed areas. White bars represent 5 µm. (D) Monopolar cells (from A, B) expressing the indicated mCherry-MKlp2 were scored as cells with polarized mCherry-MKlp2 at the cell cortex (blue), polarized with MPC completion (red) or failed MPC without mCherry-MKlp2 localization at the cell cortex but only at the monopolar spindles (green). The average percentages based on three independent experiments (total n>100 per condition, +/− standard deviation) are shown. (TIF) [file pone.0064826.s007.tif]

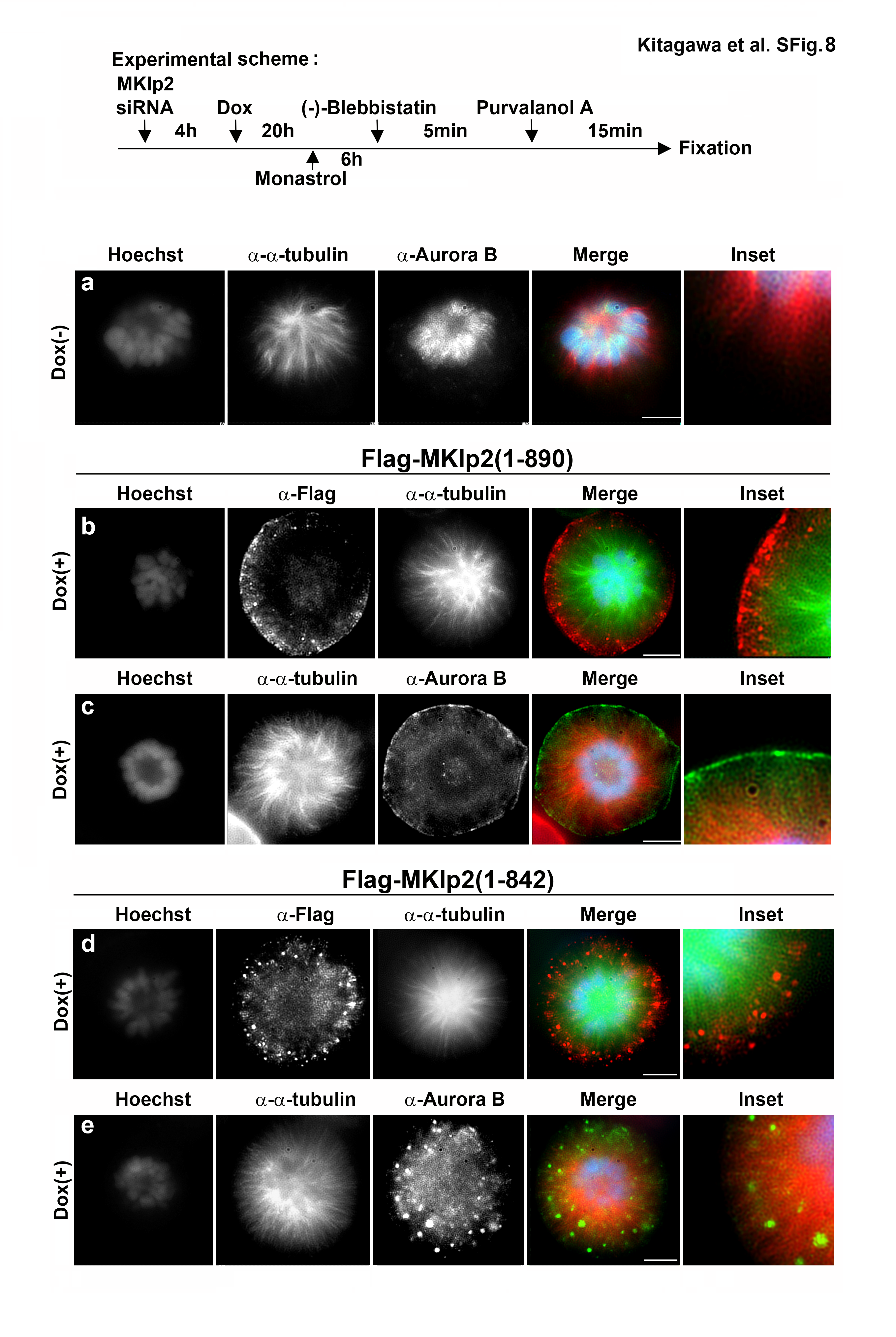

Supplement: Figure S8 — Furrowing in general is not a prerequisite for the cortical localization of MKlp2 and Aurora B. Immunofluorescence analysis. HeLa cells were transfected with MKlp2 siRNAs (50 nM) to deplete endogenous MKlp2 and treated with Dox (5 µg/ml) to express the indicated Flag-MKlp2. Next, 20 h later, cells were treated with Monastrol (100 µM) for 6 h and briefly treated with myosin-II inhibitor (–)-Blebbistatin (100 µM) for 5 min to block furrowing activity, which was then followed by purvalanol A (30 µM) treatment for 15 min. Subsequently, cells were fixed in ice-cold methanol and evaluated for the cortical localization of MKlp2 using the indicated antibodies. Note that (–)-Blebbistatin did not prevent Flag-MKlp2(1-890) and Aurora B from localizing to the cell cortex (panels b, c). Furrowing activity was inhibited; therefore, MKlp2 and Aurora B localized to the cell cortex in a non-polarized manner (panels b, c). The polarization of microtubules and the cell cortex was also completely inhibited (panels b, c). In contrast, Flag-MKlp2(1-842) together with Aurora B failed to localize to the cell cortex, but they were accumulated at the end of monopolar spindles (panels d, e). Furthermore, Aurora B in MKlp2-depleted cells did not localize to the monopolar spindles or the cell cortex but retained the chromosomes (panel a), which confirms the specificity of our imaging analysis. (TIF) [file pone.0064826.s008.tif]

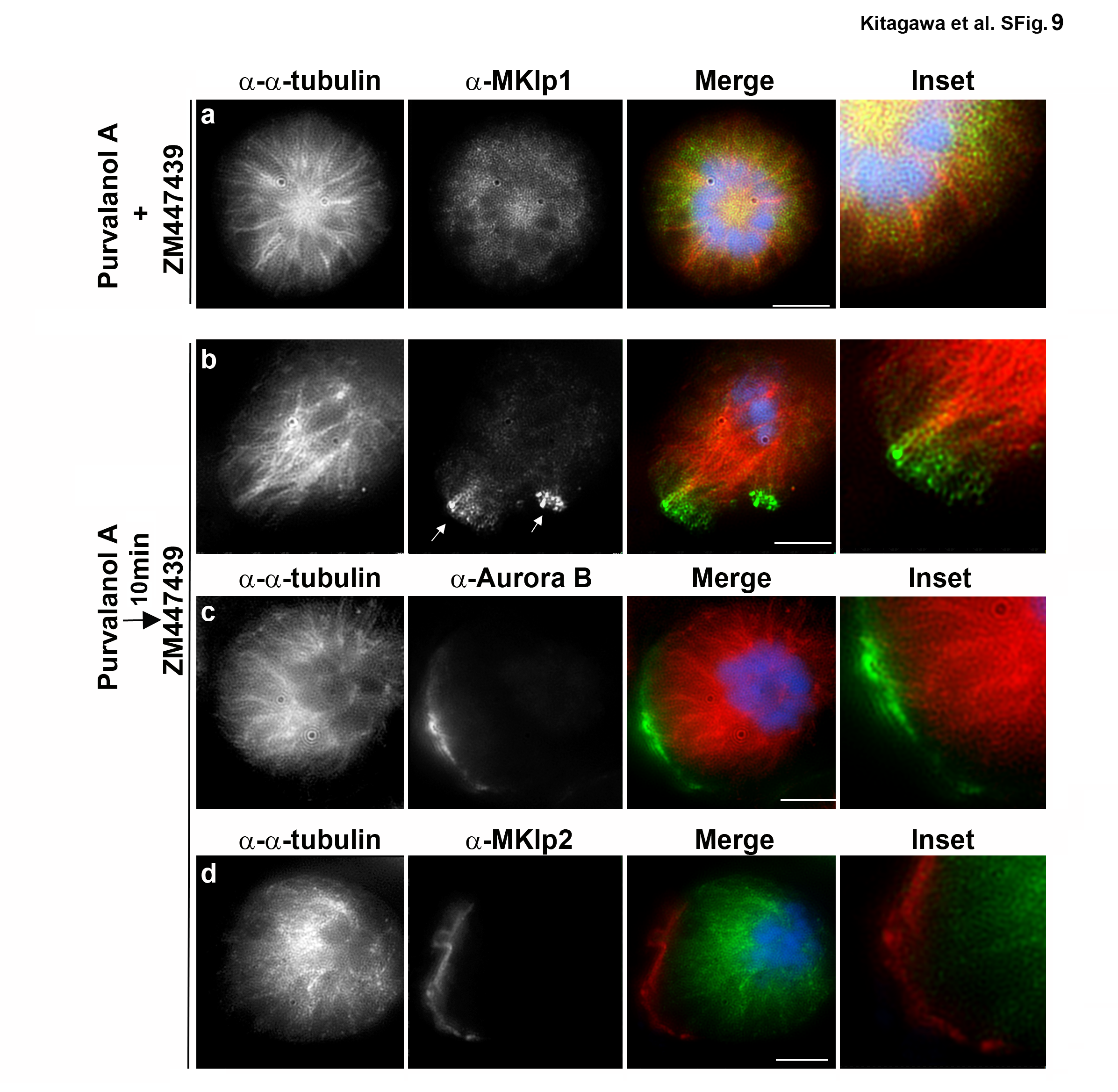

Supplement: Figure S9 — Sequential inhibition of Aurora B after polarizing cells does not alter the localization of centralspindlin at the ends of monopolar spindles. Immunofluorescence analysis. HeLa cells were subjected to monopolar cytokinesis. For panel a, purvalanol A (30 µM) and ZM447439 (2 µM) were added concurrently for 15 min. For panels b-d, ZM447439 was added sequentially after purvalanol A treatment for 10 min, and the cells were fixed 10 min after ZM447439 treatment in ice-cold methanol. Arrows (panel b) indicate MKlp1 localization at the ends of microtubules. Although the concurrent treatment of ZM447439 and purvalanol A suppressed the microtubule localization of MKlp1 (panel a) as previously shown [7], when Aurora B was sequentially inhibited after polarizing cells, MKlp1 still localized at the ends of microtubules (panel b). Moreover, Aurora B and MKlp2 largely localized to the polarized cortex. Under this condition, furrowing activity immediately ceased, and the furrows subsequently regressed (Figure 6C), suggesting that continuous Aurora B activity at the growing furrow is necessary for furrow completion. (TIF) [file pone.0064826.s009.tif]
